# Integrated Pest Management Strategies for Controlling *Phthorimaea* (*Tuta*) *absoluta*: Advances in Biological, Pheromone, and Cultural Control Methods

**DOI:** 10.3390/insects17040441

**Published:** 2026-04-21

**Authors:** Chen Zhang, Yu-Xin Wang, Xu-Dong Liu, Asim Iqbal, Qing Wang, Yu Wang

**Affiliations:** 1 Agricultural College, Jilin Agricultural Science and Technology College, Jilin 132101, China; zhangchenjl@163.com (C.Z.); wyx55445685@163.com (Y.-X.W.); liuxudong20021226@gmail.com (X.-D.L.); wangqing19691009@163.com (Q.W.); 2 Imdaad, Integrated Facilities Management Company, Street Number 1100, South Zone Jebel Ali, Dubai P.O. Box 18220, United Arab Emirates; asim_iqbal990@yahoo.com

**Keywords:** *Phthorimaea* (*Tuta*) *absoluta*, genetic variation, insecticide resistance, biological control, CRISPR/Cas system, odorant receptors, olfactory system, sex pheromone

## Abstract

The tomato leaf miner, *Phthorimaea* (*Tuta*) *absoluta*, Meyrick 1917 is recognized as a highly destructive pest, causing significant economic losses to crops in both greenhouse and open field environments across four continents: Asia, Africa, Europe, and South America. High genetic homogeneity among populations from various regions and countries indicates significant gene flow between *P. absoluta* populations, suggesting a lack of geographical barriers to dispersion. Furthermore, *P. absoluta* has developed resistance to insecticides due to target-site mutations or metabolic resistance, which enable the insect to withstand lethal doses of insecticides. To control this insect pest, the plant-mediated RNA interference (RNAi) is most promising host-induced gene silencing technique, utilized the plant’s machinery to express double-stranded (dsRNA), which triggers the RNAi pathway in *P. absoluta*. Due to thermal tolerance, the *P. absoluta* has increased its area of invasion by 600 km per year over 9 years. Female *P. absoluta* releases pheromones that are recognized by males with a sophisticated olfactory circuit on their antenna. Pheromone binding proteins (PBPs) play a crucial role in mate recognition and attraction, and their expression peaks during courtship, specifically around 6:00 a.m. Given its potential to significantly alter the insect genome, clustered regularly interspaced short palindromic repeats (CRISPR) and CRISPR-associated protein 9 (Cas9) offer a revolutionary strategy to control *P. absoluta*. Furthermore, this pest has developed remarkable adaptations to survive on unfavorable hosts by secreting specific proteins from its salivary glands that detoxify plant defenses. Insecticide resistance is likely the cause of field control failures of *P. absoluta*. Biological control, sex pheromone traps, and cultural control are the most promising approaches to address insecticide resistance resulting from these failures. Therefore, the implementation of integrated control programs and appropriate resistance management strategies is necessary to keep *P. absoluta* infestations under economic damage thresholds.

## Simple Summary

The tomato leaf miner, *Phthorimaea* (*Tuta*) *absoluta* Meyrick 1917, is a significant pest that affects tomato crops globally. Effective control of this pest typically involves a combination of integrated pest management (IPM) strategies, including the use of natural predators, sex pheromone traps, and crop rotation practices, and their combined application tends to yield significant results. Recent research highlights the potential for enhancing these traditional approaches through the incorporation of novel technologies, such as genetic control techniques, to achieve more sustainable and environmentally friendly pest management solutions.

## 1. Introduction

The tomato leaf miner, *Phthorimaea* (*Tuta*) *absoluta* (Meyrick, 1917), is a major pest affecting solanaceous crops, particularly tomatoes, and has become a global threat due to its rapid spread and high reproductive potential [1]. Native to South America, *P. absoluta* has expanded its range to regions including Europe, Africa, and Asia, causing severe economic losses in tomato production [2]. Its high reproductive potential, wide host range, and strong adaptability enable severe infestations, often causing yield losses in both greenhouse and open-field systems, making it a major concern for global food security and agricultural sustainability [3,4,5,6,7]. Implanting effective and efficient management strategies to control insect pest outbreaks is a significant challenge, as it requires a comprehensive understanding of the pest’s biology and ecology [8]. For over a decade, population genetics has traced insect pest histories and the role of genetic variation in their success [9,10]. A key strategy in managing pest outbreaks is reconstructing their spread to prevent further migration. For example, *Drosophila suzukii*, discovered in 2008, rapidly spread across the USA, emphasizing the need for early detection and management [11]. Moreover, genetic variation within insect pest populations is critical driver of their success, rapid spread, and adaptation to environmental stressors [12].

The ecological success of *P. absoluta* is supported by multiple interacting biological and environmental factors [7,13,14,15]. Its reproduction relies on sophisticated pheromone-mediated communication [16], while its broad host range enhances survival and spread.

For the development of a sustainable pest management tool, it is essential to identify the movement pathways of specific insect pest species to reduce the likelihood of their introduction into new environments [17]. One promising strategy for controlling *P. absoluta* is plant-mediated RNA interference (RNAi), a biotechnological approach designed to target specific genes in pests, disrupting their development and reducing damage [18]. Despite its potential, RNAi faces significant challenges which are discussed in this review article. Compounding these challenges is the widespread insecticide resistance seen in *P. absoluta* populations, particularly mechanisms involving target-site mutations and metabolic detoxification processes [19,20]. The spread of resistance across countries has led to a variety of country-specific resistance mechanisms, making the management of this pest even more difficult. To address these challenges, integrated pest management (IPM) strategies combining biological, pheromone, and cultural control methods are essential.

This review examines the ecological and genetic factors contributing to the spread and resistance patterns of *P. absoluta*. It highlights the importance of understanding the pest’s genetic diversity, its rapid expansion, and the evolving resistance to insecticides. The review also discusses the role of plant-mediated RNAi as a potential biotechnological solution, outlining its current status, and limitations, in the sustainable management of *P. absoluta*.

## 2. Review Methodology

**Figure 1:**
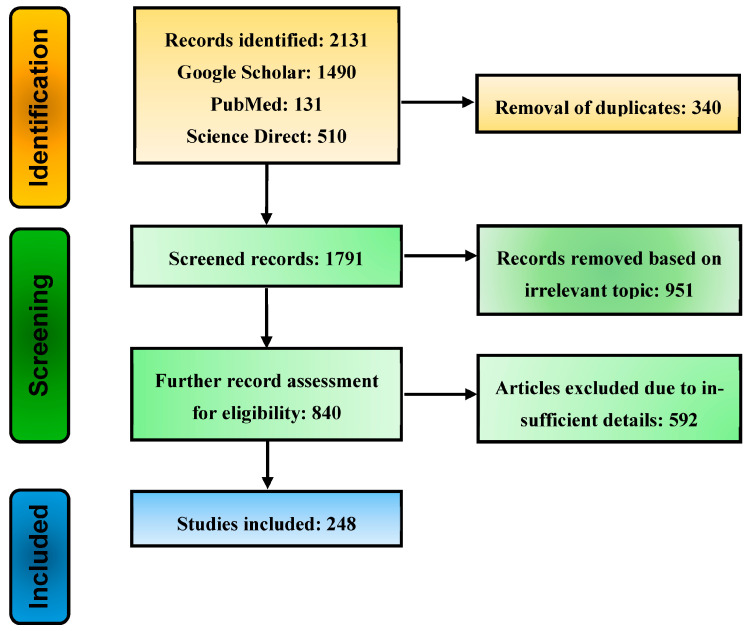
The systematic screening of relevant literature was meticulously conducted using PRISMA guidelines, with studies retrieved from prominent scientific databases.

**Figure 2:**
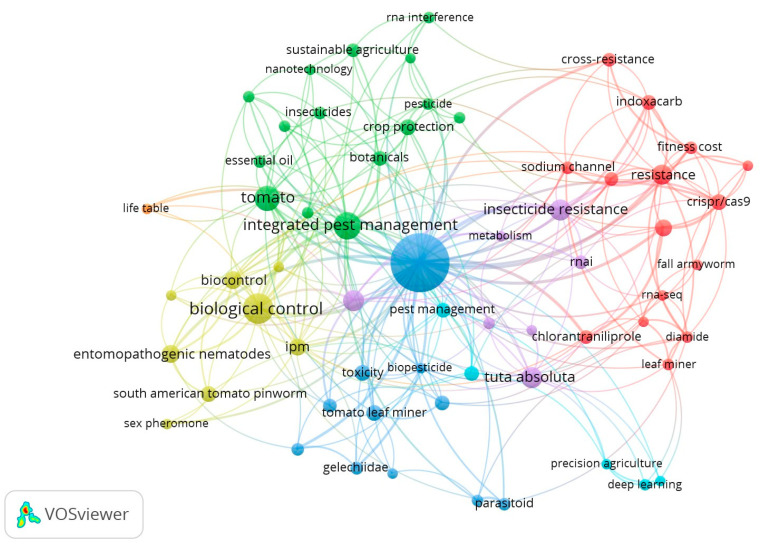
A keyword co-occurrence map demonstrating the clustering of keywords frequently used in the literature (created with VOS viewer-version 1.6.19). The size of each node indicates the frequency of keyword occurrence, with larger nodes representing higher frequencies. The connecting lines represent the correlations between keywords.

**Figure 3:**
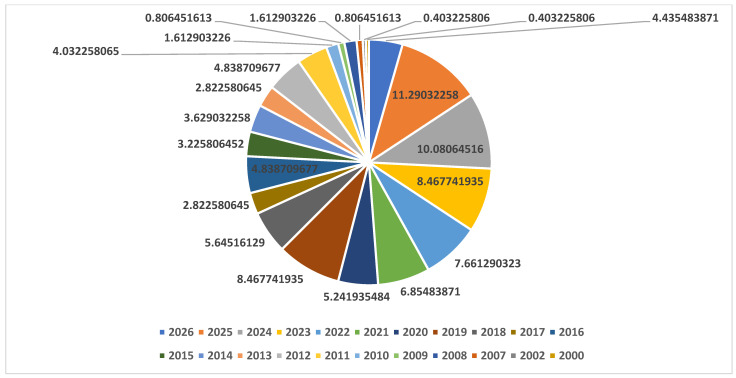
Percent contribution of research articles published during respective years.

A comprehensive literature search was performed running a query comprising keywords and Booleans: (“*Tuta absoluta*” OR “*Phthorimaea absoluta*”) AND (“insecticide resistance” OR “integrated pest management” OR “CRISPR/Cas system” OR “genetic variation”) across multiple databases, e.g., Google Scholar, PubMed, and Science Direct. The pee-reviewed article published between 2000 and 20 March 2026 were retrieved. A wide range of retrieved articles underwent a systematic selection process in line with PRISMA guidelines [21]. A total of 2131 records were retrieved from all databases, and those remained in 1791 after the exclusion of duplicates. The initial screening, based on titles and abstracts, resulted in the remaining 840 articles. Moreover, 592 records, including conference proceedings and studies with irrelevant titles and abstracts, were found short of the eligibility criteria, and 248 records were used to extract information for this review (Figure 1). Clustering and co-occurrence of keywords in the selected articles were analyzed using VOSviewer (version 1.6.19), as depicted in Figure 2. A total of 93 numbers with five distinct clusters of keywords showing most frequently, integrated pest management, biological control, *Tuta absoluta*, insecticide resistance, and CRISPR/Cas9 among all. Further bibliometric parameters included the number of publications in respective years. About 54% of the selected retrieved articles were published between 2020 and 2026, indicative of the research advancement in the prospect of the topic (Figure 3). The information gathered from the selected literature focused on the ecological, genetic, and resistance patterns of *P. absoluta*, as well as the mechanisms contributing to its rapid spread and resistance to insecticides. Additionally, the review examined the potential of plant-mediated RNA interference (RNAi) as a biotechnological solution, highlighting its current applications, limitations and future prospects in pest management. The illustrations used in this review article were created with BioRender (https://app.biorender.com).

## 3. Genetic Diversity and Resistance Patterns Across Geographic Populations

### 3.1. Taxonomy

The tomato leaf miner, *Phthorimaea* (*Tuta*) *absoluta*, Meyrick 1917 belongs to the order Lepidoptera (Family: Gelechiidae) and is native to South America [22]. As a potential insect pest of tomato, *P. absoluta* was reported for the first time in Argentina, Bolivia, Brazil, Chile, Colombia, Peru, Paraguay, and Uruguay [23,24]. In Eastern Spain, it was identified in 2006 [25], and it was first discovered in Africa in 2008 [26,27]. Then it invaded Europe, the Middle East, South Asia (India), and north, east, and West Africa [28,29,30,31,32]. The presence of *P. absoluta* was first detected in North Africa in Tunisia and Morocco in 2008 [33,34]. In West Africa, it was identified in Niger and Nigeria in 2010 [35], and later in Senegal in 2012 [36]. In East Africa, the pest was reported in Sudan (2010) [37] and Ethiopia in 2012 [38], followed by Kenya (2014) [39], Tanzania (2014) [40], and Uganda in 2015 [26]. In Southern Africa, *P. absoluta* was confirmed in Zambia [41] and South Africa in 2016 [41]. After this, it was discovered in the Kathmandu Valley in 2016 and spread to the nearest districts, including Kavrepalanchowk, Dhading, and Nuwakot [42]. Pandey, Bhattarai [7] reported that in Nepal, *P. absoluta* is recognized as a highly destructive pest that can cause up to 100% loss in a commercial tomato (*Solanum lycopersicum* Linnaeus, 1753) farm in Kathmandu. In addition, tomato, *P. absoluta* infests many other Solanaceous plants, such as potato (*Solanum tuberosum* L.), eggplant (*Solanum melongena* Linnaeus, 1753), pepino (*Solanum muricatum* Aiton, 1789), and African nightshade (*Solanum nigrum* Linnaeus, 1753) [43,44,45].

*P. absoluta* is recognized as a highly destructive pest that imposes significant economic losses on tomato. In both greenhouse and open-field environments, unchecked infestations of *P. absoluta* can result in yield reductions of 80–100% [46]. Accurate classification enables the precise identification of insect pests, which is crucial for developing targeted, eco-friendly control measures and minimizing economic losses in agriculture [47]. The classification of *T. absoluta* has been problematic, with this pest originally placed in the genus *Phthorimaea* Meyrick, 1902, a “catch-all genus for similar species. The boundaries of the tribe Gnorimoscheminis, to which *T. absoluta* belongs, have been unstable, leading to confusion in its classification [48]. Corro and Metz [48] constructed a phylogenetic tree using 22 morphological characters from species within the genera *Phthorimaea*, *Scrobipalpuloides*, and *Tuta*, resulting in a single, most parsimonious tree that groups *T. absoluta* with *Phthorimaea operculella* Zeller, 1873, and confirms the validity of the genera *Tuta* and *Scrobipalpuloides* within the tribe. Furthermore, *T. absoluta* should be reinstated under the genus *Phthorimaea*, with a new combination proposed for *Phthorimaea chiquitella* Busck 1910. Therefore, Corro and Metz [48] resolved the long-standing taxonomic uncertainty around *T. absoluta* by using a rigorous cladistics approach ultimately enhances pest control efforts.

### 3.2. Detection of Genetic Variation

Genetic variability in insect plays a significant role in the success, spread, and adaptation of insect pests [49]. In Tunisia, *P. absoluta* specimens were collected from infested tomato leaves across the northern, central, and southern regions. The study revealed low genetic diversity within pest populations in the selected regions, attributed to the founder effect, subsequent population expansion, and significant gene flow between populations. These factors have contributed to the genetic homogenization of *P. absoluta* across the country. Although various haplotypes were observed in some markers, the overall genetic variation remains markedly low [50]. Li, Fu [51] explored the broader understanding of invasive *P. absoluta* adaptation to new environments and spread across different regions of China with varying levels of genetic diversity. The larvae were collected from tomato fields in Xinjiang and Yunnan, China, and results indicated that the complete mitogenome of *P. absoluta* is 15,298 bp for the individual from Xinjiang and 15,296 bp for the individual from Yunnan, both of which are longer than the reported mitogenome from Spanish population (15,290 bp). However, higher genetic diversity, as measured by mitochondrial markers, cytochrome c oxidase subunit 2 (cox2), ATP synthase subunit 6 (atp6), NADH dehydrogenase subunit 1 (nad1), and NADH dehydrogenase subunit 5 (nad5), was observed in the Yunnan population compared to the Xinjiang population. Furthermore, using the mitochondrial cytochrome oxidase I (mtCOI) marker, high genetic homogeneity among the endosymbionts of *P. absoluta* populations collected from Iran and Turkey was observed, revealing a 100% prevalence of *Pantoea* and *Wolbachia* in these populations [52]. Javal, Ndiaye [53] investigated the genetic structure of *P. absoluta* populations in Africa. Results indicate that the African population shows high genetic homogeneity. Furthermore, *P. absoluta* in Africa likely originated from a small number of introduction events or from multiple introductions from genetically similar source populations rather than from numerous diverse sources. Wang, Tian [54] revealed a new invasion of *P. absoluta* into Gansu and Inner Mongolia, indicating the ongoing expansion of this pest. Additionally, the authors used the biological markers mtCOI and mitochondrial cytochrome oxidase I (mtCOII) and found high genetic homogeneity in the *P. absoluta* population both in China and worldwide. However, some genetic variability was observed in southern China, especially in Yunnan.

### 3.3. Resistance Patterns Across Geographic Populations

Understanding resistance patterns across different geographic populations of *P. absoluta* is crucial for designing effective and sustainable pest management programs. In this context, Zibaee, Mahmood [55] investigated organophosphate and pyrethroid resistance in *P. absoluta* from Iran. In *P. absoluta*, resistance to organophosphates arises from the A201S mutation in the ace1 gene, which results in an alteration in the structure of the acetylcholinesterase enzyme. Furthermore, substitution of alanine with serine alters the enzyme’s active site, causing a reduction in the binding affinity of organophosphates to acetylcholinesterase. The pyrethroid resistance in *P. absoluta* involves mutations in the sodium channel (kdr) genes, including L1014F, M918T, and T9291, which alter the channel’s structure and ultimately prevent the insecticide from binding to its target site. Haddi, Berger [56] identified three kdr/super-kdr mutations (M918T, T929I, and L1014F) in the para gene, specifically in the IIS4-IIS6 region of the sodium channel in *P. absoluta*, that confer resistance to pyrethroid insecticides such as lambda-cyhalothrin and tau-fluvalinate. Haddi, Berger [57] also collected strains from Brazil and Europe and identified a mutation in the ace1 gene of *P. absoluta* associated with organophosphate resistance, in which alanine at position 201 is replaced by serine. Therefore, this results in reduced sensitivity of the acetylcholinesterase enzyme to chlorpyrifos. Metabolic enzymes, such as glutathione S-transferases (GSTs) and esterases (ESTs), play a crucial role in the resistance mechanisms of *P. absoluta*. GSTs bind insecticides and catalyze their conjugation with glutathione, thereby neutralizing them and reducing their toxicity to *P. absoluta*. Furthermore, EST activity in this insect is increased when exposed to insecticides containing ester bonds, facilitating their hydrolysis and subsequent detoxification, thereby reducing their toxicity [58,59].

The resistance in *P. absoluta* is also associated with intensive pesticide use. In this context, Lewald, Tabuloc [60] conducted whole-genome sequencing on individuals from three distinct geographic regions of Latin America (Andes, Central, and North clusters). Results indicated that the Andes population showed resistance to pyrethroid, which are commonly used in this region. The *P. absoluta* population showed increased allele frequencies at resistance loci, particularly at the sodium channel gene. Furthermore, the overexpression of GST, EST, and cytochrome P450 (CYP450) enzymes in the Andes population has become a significant factor in pyrethroid resistance. Whereas the central and north *P. absoluta* populations exhibited lower levels of resistance than the Andes cluster due to less intensive pesticide use in those areas. Moreover, the identification of resistance-related loci in these insects that show signatures of positive selection indicates that insecticide use continues to exert selection pressure on these populations. Authors also found less pronounced changes in detoxifying enzyme activity in the insect populations of the central and north clusters.

## 4. Dynamics of *Phthorimaea* (*Tuta*) *absoluta* Spread in Invaded Areas

For the development of a sustainable pest management tool, it is essential to identify the movement pathways of specific pest species to reduce the likelihood of their introduction into new environments [61,62]. Central Chile is the sole source of *P. absoluta* introduction into Europe, as revealed by genetic markers [63]. Between the 1960s and 1980s, this pest spread from its native range in the Peruvian central highlands to the Latin American regions, including Argentina, Bolivia, Brazil, Chile, Colombia, Ecuador, Panama, Paraguay, Peru, Uruguay, and Venezuela [25]. Table 1 summarized the geographic distribution timeline of *P. absoluta* across various regions. Roques, Auger-Rozenberg Roques, Auger-Rozenberg [62] reported that after invading Europe, *P. absoluta* increased its area of invasion by 600 km per year over 9 years. Currently, in China, many scientists have reported its invasion in Yunnan on *Solanum indicum* (Linnaeus, 1753) [64], in Xinjiang on *S. melongena*, *S. tuberosum*, and *S. nigrum* [65]. The female *P. absoluta* prefers to oviposit on leaves, and after hatching, neonates penetrate various plant parts, creating distinctive galls and galleries [66]. In tomato leaves, mining within the mesophyll reduces photosynthetic capacity, thereby decreasing productivity, which may cause necrosis due to disrupting overall plant development [67]. Additionally, galleries within fruits can facilitate the invasion of secondary pathogens, leading to fruit rot [68,69].

### Factors Influence Phthorimaea (Tuta) absoluta Invasion

The *P. absoluta* invasion is strongly influenced by abiotic factors, which shape its population dynamics and spread [15]. Temperature is the primary abiotic factor that acts as an ecological filter, determining whether the pest can establish itself in a new environment [85]. In this context, Machekano, Mutamiswa [15] performed a thermal tolerance assay on adults and larvae of *P. absoluta*. Results indicated that larvae were more heat-tolerant, with a higher critical thermal maximum (CTmax) than adults (*p* < 0.01). In contrast, the adults were more tolerant of cold, with a significantly lower (*p* < 0.001) critical thermal minimum (CTmin) than larvae. Larvae and adults differ in their temperature tolerance, reflecting the different physiological needs and adaptations of each. The *P. absoluta* larvae maintain elevated expression levels of heat shock proteins (HSPs), which function as molecular chaperones to prevent protein denaturation and aggregation under heat stress, thereby protecting the cell from damage [86]. In addition to larvae, pupae of *P. absoluta* are also susceptible to fluctuations in environmental temperature. When the pupae were exposed to a constant temperature of 11 °C for thirty days, 47% of the pupae emerged. However, when the temperature was lowered to 10 °C for sixty days, the emergence rate decreased significantly to just 12%. Furthermore, no adult emergence was observed when the pupae were subjected to an even lower temperature of 5 °C [87]. Additionally, rainfall directly influences mortality rates in *P. absoluta* in such a way that excessive moisture can disrupt the pest’s life cycle by delaying mating and oviposition [88]. Regarding biotic factors, the spread of *P. absoluta* is facilitated by the availability of wild and cultivated Solanaceous plants, which serve as its hosts. In Botswana, wild hosts such as *Solanum coccineum* (Linnaeus, 1753), *Solanum supinum* (Jacquin, 1760), and *Solanum aculeatissimum* (Jacquin, 1760) have been identified as crucial dispersal drivers for this insect pest [15].

## 5. Ecology

### 5.1. Mate Attraction

**Figure 4:**
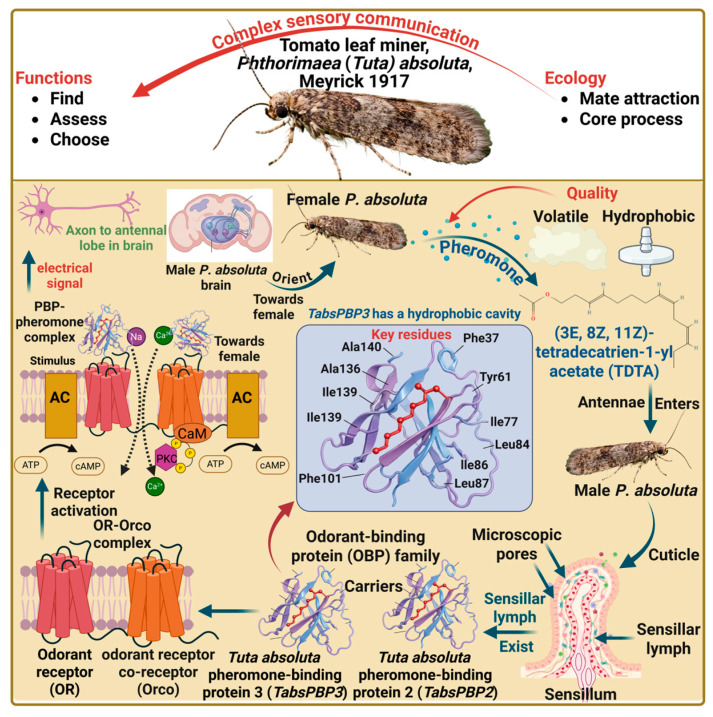
Mechanistic insights into *Phthorimaea* (*Tuta*) *absoluta* mate attraction.

Mate attraction in insects is a core part of their ecology, involving complex sensory communication to find, assess, and choose mates [89,90,91]. The odorant-binding proteins play a crucial role in the insect olfactory system, which is essential for odor discrimination [92]. The insect odorant-binding proteins (OBPs) are small, amphipathic, water-soluble, globular proteins containing about one hundred and fifty amino acids and made up of six alpha-helical domains that interact with each other by disulfide bridges [93,94]. The female *P. absoluta* releases a volatilized, hydrophobic pheromone molecule, (3E, 8Z, 11Z)-tetradecatrien-1-yl acetate (TDTA), which enters the male’s antenna through microscopic pores in its cuticle, reaching the sensillar lymph [92]. Within this lymph, the *Tuta absoluta* pheromone-binding protein 2 (TabsPBP2) and *Tuta absoluta* pheromone-binding protein 3 (TabsPBP3), specific subgroups of the larger OBP family, act as carriers. They solubilize and transport the TDTA to olfactory receptors (ORs) located on the membranes of receptor neurons [92,95]. The TabsPBP3 protein, in particular, has a hydrophobic cavity consisting of key residues, including Phe37, Tyr61, Ile77, Leu84, Ile86, Leu87, Phe101, Ala136, Ile139, and Ala140, which facilitate the binding of TDTA. The PBP-pheromone complex interacts with specific ORs and a co-receptor, Orco, triggering receptor activation. This activation sends an electrical signal through the axon to the antennal lobe in the brain and ultimately causes the male to orient toward the female [96] (Figure 4). Ou, Li [92] revealed that the TabsPBP3 expression peaks during courtship, specifically around 6:00 a.m., aligning with the timing of mating activity in the field. Furthermore, the TabsPBP3 is also highly expressed in the female pheromone gland ovipositor and may play a role in regulating adult mating behavior. The thirty-three OBP genes are also identified in the male antennae of *P. absoluta*, including *Tuta absoluta* general odorant-binding protein 1 (TabsGOBP1), *Tuta absoluta* general odorant-binding protein 2 (TabsGOBP2), *Tuta absoluta* pheromone-binding protein 1a (TabsPBP1a), *Tuta absoluta* pheromone-binding protein 1b (TabsPBP1b), and *Tuta absoluta* pheromone-binding protein 1c (TabsPBP1c) [92]. However, the clustered regularly interspaced short palindromic repeats (CRISPR) and CRISPR-associated protein 9 (Cas9) represent the most promising tools for knocking out the TabsPBP2, and TabsPBP2 genes in *P. absoluta*, thereby impairing the perception of sex pheromone TDTA. The female *T. absoluta* is also capable of auto-detecting its own pheromone, TDTA, which is weaker than that perceived by the males [97]. Moreover, the virgin females showed stronger responses than the mated females [97]. The mate choice is a fundamental behavior of insects in which individuals prefer mates that exhibit characteristics of higher reproductive quality [98]. The female *P. absoluta* prefer young, virgin, and heavy weight males for enhancing their longevity and reproductive performance [98]. Because young, virgin and heavier males produce larger and more nutrient-rich spermatophores during mating [99]. These nutrients contribute to the female’s health, increasing her longevity and reproductive performance [100,101,102].

Currently, the chemical industry is currently focused on producing synthetic pheromone lures that emit pheromones consistently and over extended periods [103]. Chermiti and Abbes [104] performed an experimental trial of installing thirty-two traps per hectare in a five-hectare field of open-field tomato crops in Kairouan city of Tunis. Results indicated that the “superdosed” TUA-Optima^®^ pheromone lures, containing 0.8 mg of synthetic pheromone, were more attractive to males of *P. absoluta* compared to the standard pheromone (0.5 mg), and are recommended for use in areas with high populations of *P. absoluta*. In Khyber Pakhtunkhwa province of Pakistan, Sadique, Sadique, Ishtiaq [105] conducted a study for two consecutive years (2020–2021) to compare the efficiency of pheromone-based traps and sticky pads in capturing male *P. absoluta* adults. The results indicated that delta traps with rubber septum pheromone lures, containing a mixture of (3E,8Z,11Z)-3,8,11-tetradecatrienyl acetate and (3E,8Z)-3,8-tetradecadienyl acetate in a 90:10 ratio, along with black sticky pads, together offer the most effective method for managing *P. absoluta*. In the United Arab Emirates, Sabbahi and Azzaoui [106] also revealed the higher efficiency of sticky traps with a mean number of *P. absoluta* adults (70.44 ± 4.57 captures/trap/week), compared to delta traps (55.94 ± 4.77 captures/trap/week) and water pan traps (18.63 ± 1.49 captures/trap/week).

CRISPR/Cas9 offers important practical advantages for next-generation pest management because this technology enables more targeted and species-focused control than conventional broad-spectrum pesticides [107]. CRISPR/Cas9 allows precise genome modification that can be used either to suppress pest traits or to improve beneficial insects used in biological control [108]. Furthermore, this tool may strengthen integrated pest management by improving efficacy, reducing reliance on chemical pesticides, and potentially lowering unintended impacts through better target specificity [109,110,111]. Their practical promise is therefore substantial, especially for the future development of sustainable and biologically informed pest-control systems [108].

Despite these advantages, CRISPR/Cas9 still faces major practical limitations that restrict their field deployment. CRISPR/Cas9 faces operational barriers, including transformation inefficiency, the need to optimize editing systems separately for each species, challenges in identifying suitable target genes, and the mass-rearing constraints associated with approaches such as the precision-guided sterile insect technique [112,113,114]. CRISPR/Cas9 requires careful biosafety assessment focused on off-target effects, environmental persistence, non-target exposure, migration risks, and possible ecological disruption [115,116,117,118,119,120]. Furthermore, this technique faces significant limitations such as repeated species-level optimization, specialized delivery systems, transformation efficiency challenges, mass-rearing needs, and regulatory testing that may increase implementation costs and delay large-scale adoption [108].

### 5.2. Host Range

Studying the host range of *P. absoluta* infestation is critical for developing effective agricultural pest management strategies and preventing significant economic losses [43]. In fifteen randomly selected villages in four leading tomato-producing districts, such as Arumeru, Kilolo, Lushoto, and Mvomero, the solanaceous crops and wild plants of Tanzania were observed for *P. absoluta* infestation. Results indicated that in the tomato field, mine density ranged from 0.1 to 4.4 mines per leaf, and the percentage of fruits damaged by *P. absoluta* ranged from 0 to 41%. Whereas, in eggplant fields, the mine density was 0 to 2.7 mines per leaf, but fruits were not affected by *P. absoluta*. In potato and African nightshade, the mine density ranges from 0 to 0.3 and 0 to 0.08 mines per leaf, respectively [43]. Archives and Blog [121] also revealed that the tomato and African nightshade are identified as the most suitable plants for *P. absoluta* invasion. Host plant selection affects the biological parameters in insect pests [122]. In this context, Negi, Sharma [123] observed that, under controlled laboratory conditions (25  ±  0.5 °C temperature, 70  ±  5% relative humidity and 12 h light: 12 h dark photoperiod), *P. absoluta* exhibited the most rapid growth on tomato leaves, with the slowest growth occurring on potato leaves. Population growth parameters, doubling time, weekly multiplication rate, intrinsic rate of increase, and net reproductive rate were highest in tomato leaves. These findings suggest that tomato is the primary and most suitable host of *P. absoluta*. A recent study by Li, Cai [124] indicated the poor adaptability of *P. absoluta* to tobacco (*Nicotiana tabacum*, Linnaeus, 1753) in terms of its growth and reproduction, compared to tomato. Furthermore, when fed on tobacco leaves, the salivary glands of *P. absoluta* larvae secrete certain proteins, such as trypsin, B5 V51-1498, and Ta74, that could facilitate the insect to adapt to the less favorable host by enhancing its ability to process or neutralize the plant defense, thus enabling it to survive and feed.

### 5.3. Multivoltinism

*P. absoluta* is currently considered a key limiting phytosanitary factor affecting the global Solanaceous crop value chain due to multivoltinism [3,125]. Multivoltinism in *P. absoluta* is regulated by thermal accumulation, and this pest requires 799.1 accumulated degree-days above a developmental threshold of 5.268 °C to complete one full generation [126]. These thermal requirements were subsequently applied to climatic data from different regions of Egypt to predict the annual number of generations. In warmer regions of Egypt, such as Qena, the highest predicted voltinism was exhibited, with approximately 9.52 generations per year. In contrast, Giza showed an intermediate value of approximately 8.05 generations per year, while the cooler region of Mersa-Matrouh recorded the lowest values, with nearly 7 generations per year [126]. This temperature-driven generational turnover of *P. absoluta* can be used to improve timely pest management.

### 5.4. Life Cycle

The insect life cycle is a key ecological factor that is significantly influenced by metamorphosis [127]. Metamorphosis is a primary driver of insect success and ecological diversity, allowing species to adapt to different environments and reduce resource competition throughout their lives [127]. *P. absoluta* undergoes complete metamorphosis and exhibits distinct life stages with unique morphological and behavioral characteristics [46,69]. The larvae is the most dangerous stage that usually affects plants leaves and also found in fruits and stems where they feed and develop, creating conspicuous mines and galleries [128]. To reduce the *P. absoluta* infestation, modeling plays a crucial role in predicting the population dynamics of this pest based on environmental parameters, thereby facilitating the development of effective management strategies [129,130]. Furthermore, the Von Foerster-based age-structured population model successfully reproduced stage-specific dynamics of *P. absoluta*, showing that eggs hatch within 4–5 days, the larval stage (comprising four instars) is the longest (approximately 20 days; about 0–70% of the life cycle), followed by a pupal stage of 4–7 days, leading to a complete life cycle of approximately 30 days [131]. Stimulation results further demonstrated that temperature strongly regulates the duration and transitions between life stages, enabling accurate prediction of peak larval abundance and allowing farmers to plan timely and efficient environmentally friendly control strategies [131].

### 5.5. Seasonal Population Dynamics

Seasonal population dynamics are a core component of population ecology because they reveal the timing and progression of pest generations during the crop season, enabling timely, efficient, and environmentally sound pest management [132]. The seasonal dynamics of *P. absoluta* are governed by the combined influence of tomato crop phenology, climatic conditions and insecticide application [133]. Infestation was highest during the fruiting stage and under warm, low-rainfall conditions, while insecticides reduced but did not fully suppress pest populations [133]. Salama, Ammar [134] also observed the seasonal fluctuations in the population of *P. absoluta* in the fields of Giza, Egypt. They found that weather variables, particularly temperature and relative humidity, are the most important drivers of pest abundance. In Beni Suef Governorate, Egypt, the seasonal peaks in *P. absoluta* populations were closely aligned with the activity of parasitoids such as *Bracon* sp., *Pteromalus* sp., and *Eulophus* sp., suggesting that these parasitoids respond to increasing pest densities and may play a crucial role in naturally suppressing *P. absoluta* populations.

### 5.6. Endosymbiont Interactions

Endosymbionts play a crucial role in the survival of insect pests, making them one of the most challenging factors in integrated pest management [135,136]. These microorganisms, which live within the insects, contribute to their fitness, nutritional needs, and resistance to environmental stressors, including insecticides [137,138,139,140]. Resultantly, the presence of endosymbionts in pest species complicates efforts to control their populations effectively within integrated pest management strategies [141]. It is difficult to define a core microbiota for a diverse group of Lepidoptera as an order [142]. Paniagua Voirol, Frago [143] compared the gut microbiomes of thirty lepidopteran species and found that Bacillaceae, Enterococcaceae, Enterobacteriaceae, Pseudomonadaceae, Staphylococcaceae were the most prevalent families, while *Bacillus*, *Enterobacter*, *Enterococcus*, *Pseudomonas*, *Staphylococcus* were the most common genera. In *P. absoluta*, symbionts such as *Acinetobacter*, *Enterobacter*, *Pseudomonas*, *Staphylococcus*, and *Wolbachia* were found in the eggs, guts, salivary glands, ovary, fat body and Malpighian tubules of this insect pest, along with some unique species like *Bacillus subtilis* and *Serratia marcescens* [144]. The specific gut bacteria *Enterobacter cloacae*, *Enterococcus gallinarum* and *Staphylococcus gallinarum* found in *P. absoluta* are essential for enhancing the adaptability of this pest to different hosts [145]. These bacteria facilitate the breakdown of plant toxins and secondary metabolites by producing enzymes like hydrolases and oxidases. These enzymes detoxify harmful compounds, enabling the insect to efficiently utilize nutrients and enhance its growth and reproduction [146]. The presence of *E. cloacae* enhances the pest’s preference and adaptation to tomatoes, while *S. gallinarum* and *E. gallinarum* assist in adapting to potato plants [145]. Moreover, these insect endosymbionts also produce enzymes such as CYP450s and GSTs that detoxify insecticides and reduce absorption. They also enhance the insect’s own detoxification pathways, promoting efficient toxin metabolism and excretion [147,148], ultimately enhancing insect pest fitness. Insects with disrupted or altered symbiotic relationships often experience reduced survival rates, making them more vulnerable to both natural enemies and environmental stressors [135]. Modern techniques like CRISPR/Cas9 and RNA interference (RNAi) allow for the targeted manipulation of these symbiotic microbes, leading to an increase in pest mortality [135]. Elston, Leonard [149] also declared that engineered symbionts can be used to manipulate insect biology and population dynamics, offering a promising, sustainable pest-control approach. Therefore, CRISPR/Cas9 and RNA interference (RNAi) can be used to edit the microbiome by targeting specific genes in gut microbes of *P. absoluta*, either silencing gene expression or eliminating bacteria, ultimately controlling this insect pest.

## 6. Factors Influencing the Spread of *Phthorimaea* (*Tuta*) *absoluta*: Climatic and Human-Mediated Factors

Climate change is projected to significantly expand the global suitability for *P. absoluta* [150]. Gao, Feng [151] indicated that the global potential climatic suitability area for *P. absoluta* is approximately 4.80 × 10^7^ km^2^, which accounts for 35.29% of the earth’s land area, excluding Antarctica. Furthermore, over one-third of the world’s land area offers suitable conditions for the establishment and spread of *P. absoluta*. These areas are predominantly found in Africa, Asia, the Americas, and Europe, which have climates favorable to the pest’s growth, development, and reproduction. The extremely high temperatures negatively affect *P. absoluta*. Specifically, temperature around 33 °C significantly reduces the survival of the pest, as high heat stress decreases its reproduction and survival while increasing mortality [152]. Furthermore, areas around the equator, where annual average temperatures are close to 30 °C, may become unsuitable for *P. absoluta* by 2050 and 2100 due to projected temperature increases of approximately 2.11 °C [151]. This increase in temperature is expected to exacerbate hot-wet stress, further limiting the pest’s development in these regions. Guimapi, Srinivasan [153] revealed that this pest spread much faster in Asia than predicted by the stimulation model, which estimated a 20-year invasion period, but the pest reached Southeast Asia in just 10 years. The rapid spread was largely driven by international crop trade and human movement, rather than just natural flight ability. Additionally, the favorable climatic conditions support the pest’s establishment in many regions. *P. absoluta* has rapidly expanded across China, primarily through two dispersal corridors originating in Xinjiang and Yunnan, with human-mediated transport, such as the movement of infested tomato plants, significantly accelerating its spread [154]. This expansion is coupled with a substantial shift in the pest’s climatic niche, allowing it to adapt to a broader range of temperature and precipitation conditions. The pest’s physiological adaptations, such as enhanced cold tolerance and increased supercooling capacity, enable it to thrive in colder regions of China that were previously climatically unsuitable [154]. Furthermore, the widespread use of irrigation creates artificial microclimates, which buffer against environmental extremes [155], supporting its survival in arid and temperate regions [3,15]. This combination of biological adaptability and anthropogenic modifications has allowed *P. absoluta* to colonize a wider range of agroecosystems compared to other regions. These findings underscore the need for enhanced biosecurity measures to manage its spread.

## 7. Insecticide Resistance in *Phthorimaea* (*Tuta*) *absoluta*

Insecticide resistance is likely the cause of field control failures of *P. absoluta* [156]. In Kenya, the *P. absoluta* populations exhibited high frequencies of resistance genes such as kdr and ace-1 mutations [157]. The kdr mutation is caused by point mutations in the voltage-gated sodium channel (VGSC) gene, specifically in the S6 transmembrane region of domain II of the VGSC protein. The most common mutation is a leucine to phenylalanine substitution (L1014F), which alters the sodium channel’s structure and reduces its sensitivity to pyrethroid. This region is crucial for controlling the opening and closing of the sodium channel, and the mutation prevents pyrethroid from binding effectively, thereby leading to resistance in *P. absoluta* [157]. Furthermore, the ace-1 gene encodes acetylcholinesterase (AChE), an enzyme crucial for breaking down acetylcholine in the insect nervous system [158,159]. A mutation in this gene in *P. absoluta*, particularly point mutations that lead to a single-nucleotide polymorphism (SNP), alters the enzyme’s structure, especially at the active site [160]. This reduces AChE’s affinity for insecticides like organophosphates and carbamates, allowing the enzyme to continue breaking down acetylcholine even in the presence of these chemicals [157]. As a result, the insect pest survives, and the resistant trait spreads, making insecticide treatments less effective over time [157]. Furthermore, Ong’onge, Ajene [157] also stated that after about five years of insecticide application, the pest population started developing resistance, and this trend is predicted to continue, leading to failure of the chemical control method if not managed properly.

Resistance to key insecticides has evolved dramatically in field populations of *P. absoluta*, with varying degrees of resistance across different chemical classes [156]. Pyrethroids, including bifenthrin and permethrin, exhibited only low resistance (>12.5-fold), while *Bacillus thuringiensis* and the mixture deltamethrin + triazophos similarly showed limited resistance. In contrast, chitin synthesis inhibitors (diflubenzuron, triflumuron, and diflubenzuron) exhibited high resistance levels (up to 222.3-fold), suggesting a critical failure in their long-term efficacy. Indoxacarb also showed moderate resistance, with a 27.5-fold increase compared to susceptible populations. Notably, the emergence of resistance was strongly linked to insecticide use patterns, with a disproportionate reliance on chitin synthesis inhibitors exacerbating resistance development [156]. Furthermore, the insecticide resistance against *P. absoluta* has been reported from various countries such as China [161,162], Kenya [157], Latin America [60]. Zhang, Li [162] utilized insecticide efficacy monitoring as a basic tool for proactive evidence-based resistance management. They investigated the susceptibilities of seven populations (Gansu, Guizhou, Inner Mongolia, Shanxi, Sichuan, Xinjiang, Yunnan) of *P. absoluta* across China against six insecticides (*Bacillus thuringiensis*, chlorpyrifos, chlorantraniliprole, emamection benzoate, indoxacarb, and spinosad. The results indicated that *P. absoluta* exhibited the highest resistance to chlorpyrifos and chlorantraniliprole in Shanxi and Yunnan, while most populations showed low resistance to *B. thuringiensis* emamection benzoate, indoxacarb, and spinosad, highlighting the need for rational insecticide use to manage emerging resistance. Guedes, Roditakis [163] revealed that the development of insecticide resistance is relatively fast in this species in South America and Europe due to altered target-site sensitivity and/or enhanced detoxification. Therefore, the implementation of integrated control programs and appropriate resistance management strategies is necessary to keep *P. absoluta* infestations under economic damage thresholds. Table 2 provides an overview of the country-wise insecticide resistance mechanisms in *P. absoluta*.

## 8. Other Management Strategies

### 8.1. Biological Control

Biological control is one of the components of an integrated management program through which a number of natural enemies are utilized against *P. absoluta* to keep its population below the economic threshold level [175,176]. The biological control agents include predators, parasitoids, pathogens, and nematodes are successfully utilized against insect pests [177]. Rubio, Montes [178] claimed that joint use of both predator *Macrolophus basicornis* (Stal, 1860) and *Trichogramma pretiosum*, (Riley, 1879) is a better option for decreasing the *P. absoluta* population as compared to the use of each biological control agent separately. Furthermore, the introduction of *M. basicornis* after *T. pretiosum* release significantly reduced the adult *P. absoluta* population by 28%, decreasing even more the damage caused in tomato crops compared to the use of *T. pretiosum* alone. It is necessary to determine the predators and parasitoid species to assess the effectiveness of these natural enemies for biological and integrated pest management in crops.

Akat and Bayhan [179] performed a survey to determine predators and parasitoid species of *P. absoluta* in tomato field in Diyarbakir province, Turkey and found the three parasitoids such as *Bracon didemie* (Beyarslan, 2002), *Bracon viktorovi* (Tobias, 1961), and *Nracon hebetor* (Say, 1836); and ten predators including, *Campylomma diversicornis* Reuter, 1890; *Chrysoperla carnea* (Stephens, 1836); *Coccinella septempunctata* (Linnaeus, 1758); *Geocoris megacephalus* (Rossi, 1790); *Hippodamia variegate* (Geoffroy, 1777); *Macrolophus costalis* (Fieber, 1858); *Macrolophus pygmaeus* (Rambur, 1839); *Nysius graminicola* (Lolenati, 1845); *Orius* spp. *Orius niger* (Wolff, 1804). Nearly sixty species of predators, belonging to approximately twenty-six families, have been employed as natural enemies against *P. absoluta*. Of these, fifty species have been recorded in South America, while only ten species have been documented within their invasive range, particularly in European countries [180].

The significance of integrated pest management using predators against *P. absoluta* became evident in areas that were invaded early. Current findings suggest that the commercial availability of biological control agents has played a significant role primarily in these early-invaded regions, such as southern Europe [181,182]. However, it is still too early to determine whether similar biological control practices will be effective in managing this pest in areas that have recently experienced invasion [183]. Despite this, two commercially available mirid bugs, *Nezara viridula* (Linnaeus, 1758) and *Macrolophus pygmaeus* (Rambur, 1839), have emerged as key biological control agents in Europe [184]. Pérez-Hedo, Riahi [185] also stated that the *Nesidiocoris tenuis* (Reuter, 1905), *M. pygmaeus*, and *Dixyphus Hesperus* (Knight, 1923) hold significant potential for enhancing *P. absoluta* pest management in horticultural crops. In 2020, the green lacewing, *Chrysoperla carnea* (Stephens, 1836), was first utilized as a promising biocontrol agent for *P. absoluta* and assessed its efficiency in controlled conditions and the field [186]. Laboratory results indicated that *C. carnea* consumed 36 ± 2 eggs within 24 h and 72 ± 4 eggs within 48 h, with 2% of larvae killed inside and 35% killed outside leaf galleries. Whereas, in field trials, the release of *C. carnea* reduced larval density by 4 to 6 times compared to control plots. The biocontrol plots showed lower pest density and damage, and significantly higher tomato yield compared to control plots, suggesting that *C. carnea* is an effective biological control agent for managing *P. absoluta* [186]. Future perspectives include expanding field trials, integrating with other biocontrol agents, developing commercial production methods, optimizing release timing, exploring its impact on other pests, and promoting sustainable, eco-friendly pest management strategies.

The association of parasitoids with *P. absoluta* is critically important for sustainable pest management, offering a natural, eco-friendly alternative to chemical insecticides that often fail due to pest resistance [187]. In 2019, Salas Gervassio, Aquino [188] re-examined the parasitoids of *P. absoluta* for optimized biological control in South America. They reported that over fifty species of morphospecies of Hymenoptera were associated with *P. absoluta*, but only half of these could be confirmed as parasitizing the pest. This limitation was attributed to incomplete or unreliable species identification, erroneous species names, and a lack of supporting literature [188]. The inoculative seasonal release of endoparasitoid, *Pseudapantales dignus* (Muesebeck, 1938) has proven to be an effective and reliable crop protection strategy, both in open fields and in greenhouses, as an augmentative biocontrol method [189]. In a semi-field greenhouse setup, the *P. absoluta*, *P. dingus* relative densities ranging from 2:1 to 10:5 were tested. The parasitism rates ranged from 23% to 61%, with the highest parasitism observed at a density of 10:3. At low host densities, parasitism was negligible due to reduced host localization cues. The higher parasitoid release rates resulted in decreased parasitism, potentially due to mutual interference among females, suggesting the potential of *P. dingus* for biological control, though its effectiveness varies with host density [189]. The abundance and functional diversity of natural enemies is also associated with the attractiveness of insectary plants [190]. The flowering plants, including *Achillea millefolium* (Linnaeus, 1753), *Fagopyrum esculentum* (Moench, 1802), *Lobularia maritima* (Linnaeus) Desvaux, 1814), and *Sinapis alba* (Linnaeus, 1753), were grown in proximity to tomato plants that significantly attract natural enemies, including *Aeolothrips* spp. *Coccinella* spp., Hoverflies, *M. pygmaeus*, *N. tenuis*, *Necremnus tutae* (Ribes & Bernardo, 2015), *Orius* spp. [190]. These insectary plants provide critical resources, including nectar, pollen, and alternative food sources that support the survival, fitness, and foraging behavior of these beneficials [191,192].

The host specificity of parasitoid depends on their native or invasive nature. For instance, the native *N. tutae* is polyphagous and attacks multiple non-target species in the laboratory, such as *Cameraria ohridella* (Deschka & Dimic, 1986), *Liriomyza bryoniae* (Kaltenbach, 1858), and *Leucoptera malifoliella* (O. Costa, 1836) [193]. In contrast, the invasive endoparasitoid *Dolichogenidea gelechiidivoris* (Marsh, 1975) attacks only *P. operculella*. In greenhouse trials, *N. tutae* did not show a preference between *P. absoluta* and *P. operculella*. Whereas *D. gelechiidivoris* exhibited a preference for *P. absoluta*, with a significantly higher parasitism rate on tomato plants infested with *P. absoluta* compared to *P. operculella* on potato plants [193]. The difference in host preference between the two parasitoids stems from their ecological specialization and evolutionary history with *P. absoluta*. Nematodes are also the most promising biological control agent, which belongs to the Heterorhabditidae and Steinernematidae families [194,195]. Nematodes of the genera *Steinernema* and Heterorhabditis contain symbiotic bacteria belonging to the genera *Xenorhabdus* and *Photorhabdus*, respectively, which are responsible for causing mortality to insects [196]. The infective juveniles enter hosts through natural openings and release their symbiotic bacteria that eventually kill the host [196,197]. In this context, Kamou, Papafoti [198] investigated the effects of two entomopathogenic nematode species, *Steinernema carpocapsae* (Weiser, 1955) and *Heterorhabditis bacteriophora* (Poinar, 1976), as well as their bacterial symbionts, *Xenorhabdus nematophila* (Poinar & Thomas, 1965) and *Photorhabdus luminescens* (Poinar & Thomas, 1979), against *P. absoluta* larvae. The results indicated that the *S. carpocapsae* and *H. bacteriophora* were the most effective, causing approximately ninety-eight percent mortality of *P. absoluta* larvae. Regarding the bacteria, *X. nematophila* was the most effective, causing 69% mortality in young larvae, thereby suggesting its potential as a biocontrol agent in the field following augmentative release [198]. Many studies have also demonstrated that *P. absoluta* larvae are highly susceptible to entomopathogenic nematodes, which are used as a biocontrol agent [199,200,201,202]. Table 3 summarizes the efficacy of various natural enemies of *P. absoluta*.

Plant-mediated RNA interference (RNAi) is the most promising and innovative, and sustainable pest management strategy for controlling insect pests by targeting crucial insect genes through transgenic expression [203]. In Turkey, Hashmi, Tariq [204] utilized this technique, in which transgenic tomato plants were genetically engineered to produce double-stranded (dsRNA) targeting the two key genes acetylcholinesterase 1 (AChE1) and SEC23, which are essential for *P. absoluta* survival. When feeding on transgenic plants, this insect pest ingests dsRNA, which enters the insect cell via the digestive system. The dsRNA triggers the RNAi pathway, where it is processed into small interfering RNAs (siRNAs) that bind to the mRNA of target genes, resulting in gene silencing and preventing the production of vital proteins. The silencing of AChE1 and SEC23 leads to nerve dysfunction and disrupts insect physiology, respectively, making larvae more susceptible to insecticides, such as organophosphates. As a result, higher mortality, reduced growth, and developmental abnormalities ultimately provide an effective pest control method [204]. Current practical limitations of plant-mediated RNAi remain substantial despite its promise for pest control. Field deployment is constrained by delivery inefficiency, because RNAi performance depends on sufficient dsRNA accumulation in plant tissues [205], while insects cannot amplify the RNAi signal and many species rapidly degrade ingested dsRNA through gut nucleases, reducing silencing efficiency [206,207]. Target selection is also a major challenge, as not all essential genes respond equally well to silencing, and RNAi sensitivity differs across insect groups [208,209]. In addition, nuclear transformation may produce low and unstable dsRNA expression, whereas plastid transformation, although often more effective, is currently feasible in only a limited number of crop species [210].

The large-scale application of plant-mediated RNAi is still more experimental than operational and remains largely confined to laboratory or small-scale settings, while commercialization requires broader field validation, resistance management, and crop-specific optimization [203]. Biosafety and regulatory scrutiny remain important because human safety, non-target effects, and ecological assessment must be addressed, yet these evaluations are complicated by the lack of full genomic information for many non-target organisms [211,212]. Scalability is further limited because not all crops are easily transformed and a single transgenic strategy may not control multiple pests effectively [109]. Although RNAi may have relatively low upfront development costs, the costs of large-scale commercial deployment still require careful consideration before widespread adoption [213,214].

**Table 3:** Natural enemies of *Phthorimaea* (*Tuta*) *absoluta* and their reported efficacy.

Natural Enemy	Group	Type of Control Agent	Target Stage of *P. absoluta*	Reported Efficacy/Performance	Study Condition	Region/Country	Key Findings	References
*Trichogramma achaeae*	Hymenoptera: Trichogrammatidae	Parasitoid (Egg)	Eggs	Parasitism and emergence unaffected by resistant or susceptible genotypes, but egg size influenced the proportion of female parasitoids. Fewer female parasitoids from resistant plants.	Isolation and tomato leaflets	Spain	*Solanum arcanum* negatively impacted parasitism and emergence due to high glandular trichomes.	[215]
*Necremnus tutae*	Hymenoptera: Eulophidae	Parasitoid (Larval)	Larvae (Second to third instar)	Fewer parasitoids emerged on *S. arcanum* compared to other genotypes.	Infested leaflets with larvae	Spain	*S. arcanum* and *Solanum neorickii* negatively affected parasitism performance.	
*Macrolophus pygmaeus*	Hemiptera: Miridae	Predator	Eggs and larvae	Predation lower on *S. arcanum* due to high glandular trichomes. Higher predation on *S. neorickii* and other genotypes.	Egg and larval predation on tomato genotypes	Spain	*S. arcanum* hinders predator efficacy, while *S. neorickii* allowed better predation success.	
Black Soldier Fly Oil & Neem Oil	Insect-derived/Plant-derived	Biorational (Ovicidal, Larvicidal, Antifeedant)	Eggs and larvae	Moderate ovicidal suppression (20–55% mortality); higher larval mortality (33.8–92.9%); leaf penetration deterrence and increased larval mortality in treated plants.	Semi-field screenhouse trials	Kenya	Significant egg mortality and larval mortality in both *P. absoluta* and *Spodoptera frugiperda*. Insect oil exhibited lower LC_50_ than neem oil in larvicidal bioassays.	[216]
*Neochrysocharis formosa* (thelytokous (TH) strain)	Hymenoptera: Eulophidae	Parasitoid	1st instar larvae	High parasitism rates and effective host-stinging on 1st instar larvae. Parasitism and stinging rates are higher compared to AR strain, especially in lower density settings.	Laboratory/controlled conditions	China	TH strain is more effective at controlling *P. absoluta* in early infestations due to higher preference for 1st instar larvae and efficient parasitism.	[217]
*Neochrysocharis formosa* (arrhenotokous (AR) strain)	Hymenoptera: Eulophidae	Parasitoid	1st and 2nd instar larvae	Parasitism less effective than TH strain, with host-stinging and feeding behavior being more prominent at higher densities.	Laboratory/controlled conditions	China	AR strain exhibits lower attack rates compared to the TH strain, especially when larvae are more than 1st instar.	
*Dolichogenidea gelechiidivoris*	Hymenoptera: Braconidae	Parasitoid (Larval)	1st, 2nd, 3rd, and 4th instars	Parasitized and successfully developed in all four host larval instars. Females preferentially oviposited in early instars (1st and 2nd).	Laboratory conditions at 26 ± 4 °C	Kenya	High parasitism in early instars, with significant differences between early (1st and 2nd) vs. late (3rd and 4th) instars in terms of egg deposition and cocoon formation.	[218]
*Necremnus artynes*	Hymenoptera: Eulophidae	Parasitoid (Larval	Larvae (2nd and 3rd instars)	Significant increase in longevity with buckwheat, *Fagopyrum esculentum* and sugar solution. Host-feeding was not as effective in increasing longevity	Greenhouse, laboratory conditions	Belgium	Longevity was significantly enhanced by *F. esculentum* and sugar solution. Host-feeding did not significantly increase longevity.	[219]
*Bracon hebetor*	Hymenoptera: Braconidae	Parasitoid	4th and 5th instars	The highest parasitism rates and fecundity observed on *Galleria mellonella* larvae. Lower parasitism rates for *P. absoluta* and *Phthorimaea operculella*.	Laboratory/greenhouse conditions	Faisalabad/Pakistan	Best performance on *G. mellonella*, poor performance on *P. absoluta*. Longevity and egg-laying capacity are affected by diet and host conditions.	[220]
*Nesidiocoris tenuis*	Hemiptera: Miridae	Predator	All stages	Presence significantly reduced *P. absoluta* population growth. However, exposure to lambda-cyhalothrin affected predation behavior and longevity.	Laboratory/greenhouse conditions	Alenya/France	Effective as a predator against *P. absoluta*, but its behavior and longevity are negatively impacted by chemical treatments, especially lambda-cyhalothrin.	[221]
*Dicyphus errans*	Hemiptera: Miridae	Predator	Eggs, 1st-instar larvae	Effective in preying on *P. absoluta* eggs (up to 12.4 eggs/day). Females consumed more eggs and larvae than males.	Laboratory conditions	Italy/Europe	Females consumed significantly more eggs (73.6%) compared to males (57.6%). Preference for 1st-instar larvae.	[222]
*Dolichogenidea gelechiidivoris*	Hymenoptera: Braconidae	Parasitoid (Larval)	All developmental stages	The parasitoid performed well in all regions except for coastal areas under the current climatic scenario and is predicted to improve under future scenarios.	Field conditions, climate modeling	Kenya	The fuzzy model predicted good performance across regions, with significant improvements in the Rift valley and coastal regions under future climate scenarios.	[223]
*Macrolophus pygmaeus*	Hemiptera: Miridae	Predator	Eggs, larvae	Intraguild predation (IGP) occurs when *Macrolophus pygmaeus* feeds on parasitized eggs, mainly early in the development of parasitoid larvae.	Laboratory/greenhouse conditions	Mediterranean regions	Exhibited preference for parasitized eggs in the early stages (yellow eggs), leading to reduced parasitoid survival rates.	[224]
*Trichogramma achaeae*	Hymenoptera: Trichogrammatidae	Parasitoid (Egg)	Eggs	Significant increase in pest control when combined with *M. pygmaeus*, despite some negative effects from intraguild predation.	Laboratory/greenhouse conditions	Mediterranean regions	Combining *Trichogramma* parasitoids with *M. pygmaeus* improves pest control compared to using either agent alone.	
*Stenomesius japonicus*	Hymenoptera: Eulophidae	Parasitoid (Larval)	3rd instar larvae	Effective in controlling *P. absoluta* larvae, with high parasitism rates observed when alone. However, parasitism decreased in the presence of the omnivorous predator.	Laboratory/greenhouse conditions	France	When combined with *M. pygmaeus*, parasitism rates were negatively affected by intraguild predation but still had significant pest control.	[225]
*Macrolophus pygmaeus*	Hemiptera: Miridae	Predator (Generalist)	Eggs and larvae	Strong immediate effect on *P. absoluta* eggs and larvae. However, its population was lower when in competition with *S. japonicus* as it faced intraguild predation	Laboratory/greenhouse conditions	France	*M. pygmaeus* had a stronger initial impact on pest populations, but its longer-term effectiveness was reduced by competition with *S. japonicus*.	

LC_50_, Lethal concentration that kills 50% of the population.

### 8.2. Use of Sex Pheromones

Pheromone traps are a critical, sustainable tool for managing insect pests by utilizing synthetic pheromones to attract and capture male moths [226]. Yang, Cai [227] synthesized the pheromones (3E,8Z,11Z)-3,8,11-tetradecatrienyl acetate (1) and (3E,8Z,11Z)-3,8,11-tetradecatrienyl acetate (2) in seven-step process. The synthesis begins with tetrahydropyranyl (THP)-protected 4-bromo-1-butanol, followed by alkylation with 3-butyn-1-ol, reduction with lithium aluminum hydride (LiAIH4), acetylation, oxidation to form an aldehyde, and finally a Witting reaction with phosphate salts to yield the desired pheromones. Jabamo, Ayalew [228] reported that using sex pheromone reduced *P. absoluta* damage on tomato. However, sex pheromone and insecticide resulted in enhanced effect against *P. absoluta*. In 2021, the Yili region in Xinjiang, China, was invaded by *P. absoluta* and in response, the use of sex pheromone-based control techniques proved to be an effective method for managing this pest in the region [229]. However, polygyny in the Yili population is likely to reduce the effectiveness of sex pheromone-based control methods, suggesting caution for growers relying on this technique. Because in polygyny multiple *P. absoluta* females mate with a single male, it skews the sex ratio and reduces the effectiveness of sex pheromone-based control methods. The increased number of females diminishes the disruption of mating, potentially allowing continued pest reproduction despite pheromone intervention [229]. The appropriate timing of using pheromone traps is also a key factor in successful implementation of this technique. In this context, Zhang, Zhang [230] found that *P. absoluta* responded most strongly to sex pheromone lures from 05:30 a.m. to 08:30 a.m., with 95.8% of males captured during this period. The peak response occurred at 07:30 a.m., with 80.8% of males caught, highlighting the optimal time for using sex pheromone traps, aiding the development of more effective integrated pest management strategies. The more information regarding pheromone traps performance in monitoring and management of *P. absoluta* in Table 4.

### 8.3. Cultural Control

For both open field and greenhouse tomatoes, routine monitoring and removal of infested leaves in the early stages help reduce the initial pest population. Although labor-intensive, these practices are effective and straightforward. Other cultural control methods include intercropping, crop sanitation, crop rotation, deep plowing, and proper weed management [242,243]. Rotating tomato crops with non-solanaceous crops is essential for disrupting the life cycle of *P. absoluta* and can help in reducing pest infestation. This management strategy limits the chances of pest development and spread to the next generation [242]. Moreover, enhancement of resilience towards tomato crops requires proper supplements by either incorporating organic matter, such as manure, or synthetic nitrogenous fertilizers that nurture heavy crops that naturally resist *P. absoluta* attack and subsequent damage [244,245]. Additionally, applying dustable sulfur over tomato crops negatively affects the infestation and oviposition of *P. absoluta* due to its repellent effect [246]. Intercropping, the practice of growing multiple crops together, is a crucial sustainable pest management technique that reduces insect infestations by increasing agro-ecosystem diversity [247]. Zarei, Fathi [248] reported that intercropping tomatoes with sainfoin, *Onobrychis viciifolia* (Scop. 1771) significantly disrupting pest colonization and enhacing predators (*O. niger* and *N. tenuis*) activytity. *O. viciifolia* also provides resources to these predaotrs. Additonaly intercropping boosts soil fertility, leading to higher crop yields.

## 9. Conclusions and Future Perspectives

This review highlights the importance of adopting IPM strategies to control *P. absoluta*. Effective management involves combining multiple methods such as biological control agents, including parasitoids and predators, with the strategic use of pheromone traps and cultural practices like intercropping and crop rotation. Combining these methods offers the best long-term results for pest control, minimizing the need for chemical pesticides and promoting sustainable agricultural practices. However, the variability in pest control effectiveness based on environmental conditions, pest populations, and ecological factors calls for continued refinement and local adaptation of these techniques. The ongoing development of novel approaches, including CRISPR/Cas9 and plant-mediated RNAi as genetic-based solutions, holds great potential for the future of pest management. Future field trials for *P. absoluta* management will focus on integrating RNAi with traditional methods, enhancing pheromone traps, and exploring ecological interactions and cultural practices like intercropping.

## Figures and Tables

**Table 1:** Geographic distribution timeline of *Phthorimaea* (*Tuta*) *absoluta* worldwide.

Region/Continent	Country/Area	Year of First Report	Probable Route of Spread	Major Host Crops Affected	Current Status	References
Africa	Algeria	2008	Trading of tomato fruits	Tomato (*S. olanum lycopersicum*)	Present	[70]
	Morocco	2008	Trading of tomato fruits	Tomato (*S. lycopersicum*)	Present	[71]
Asia	Israel	2009	Trading of tomato fruits	Tomato (*S. lycopersicum*)	Present	[72,73]
	India	2009	Trade, agriculture imports	Tomato (*S. lycopersicum*)	Expanding	[74]
	Tajikistan	2018	Likely through trade, spread via tomato seedlings	Tomato (*S. lycopersicum*), Potato (*Solanum tuberosum*), Sweet pepper (*Capsicum annuum*)	Established, causing severe damage to tomatoes	[75]
Europe	Spain	2006	Trading of tomato fruits	Tomato (*S. lycopersicum*)	Rapid spread across Mediterranean	[76]
	France (Including Corsica)	2008	Trading of tomato fruits	Tomato (*S. lycopersicum*), Eggplant (*Solanum melongena*)	Established populations	[77]
	Italy (Including Sicily and Sardinia)	2008	Trading of tomato fruits	Tomato (*S. lycopersicum*), Potato (*Solanum tuberosum*), Eggplant (*S. melongena*)	Widely spread	[78]
	Albania	2008	Trading of tomato fruits	Tomato (*S. lycopersicum*)	Present	[77]
	Bulgaria	2009	Trading of tomato fruits	Tomato (*S. lycopersicum*)	Present	[72]
	Greece	2009	Trading of tomato fruits	Tomato (*S. lycopersicum*)	Present	[79]
	Russia	2010	Trading of tomato fruits	Tomato (*S. lycopersicum*)	Present	[80]
Middle East	Tunisia	2009	Spread via imports	Tomato (*S. lycopersicum*)	Endemic	[77]
	Saudi Arabia	2010	Trading of tomato fruits	Tomato (*S. lycopersicum*)	Present	[81]
	Iran	2016	Likely via trade from Mediterranean regions	Tomato (*S. lycopersicum*)	Established	[82]
South Asia	Pakistan (Punjab)	2018	Likely through trade, spread via tomato seedlings	Tomato (*S. lycopersicum*)	Established in Charsadda; spreading in Rawalpindi	[82]
	Pakistan (Khyber Pakhtunkhwa)	2019	Likely via trade, urban-to-urban movement	Tomato (*S. lycopersicum*), Potato (*S. tuberosum*)	Spread in Charsadda, spreading across Khyber Pakhtunkhwa	[82]
Sub-Saharan Africa	Ghana	2017	Likely through imports	Tomato (*S. lycopersicum*)	Present	[83]
West Africa	Côte d’Ivoire (nationwide: North, South, East, West, Center regions)	2016	Likely regional spread via trade and/or natural dispersal from neighboring countries (e.g., Senegal, Ghana, Burkina Faso, Mali)	Tomato (*S. lycopersicum*); eggplant (*S. melongena*), black nightshade (*Solanum nigrum*), pepper (*Capsicum annuum*)	Fully established and widely distributed across all surveyed regions with high infestation levels	[84]

**Table 2:** Country-wise insecticide resistance mechanisms and resistance ratios in *Phthorimaea* (*Tuta*) *absoluta*.

Country	Insecticide/Active Ingredient	Class	Population/Strain	Resistance Ratio (RR)	Mechanism Identified	Method/Evidence	Remarks/Management Implication	References
Brazil	Chlorantraniliprole, Flubendiamide	Diamide insecticides	BR-GML1, BR-PSQ	>3000-fold	G4903E, I4746M mutations	Genotyping, pyrosequencing	High resistance in Brazilian strains, highlighting the importance of early resistance detection and management	[164]
	Deltamethrin	Pyrethroid	Various Brazilian populations	1.18 to 5.12	Target site mutation: L1014F, M918T, T929I	Bioassays, TaqMan diagnostic assay	High frequency of L1014F mutation, suggesting widespread resistance. Control failure in all populations.	[165]
	Alpha-Cypermethrin	Pyrethroid	Various Brazilian populations	1.27 to 11.10	Target site mutation: L1014F, T929I	Bioassays, TaqMan diagnostic assays	The T929I mutation appears to provide selective advantage in some populations. Widespread resistance.	[165]
	Permethrin	Pyrethroid	Various Brazilian populations	1.26 to 5.27	Target site mutation: L1014F, M918T, T929I	Bioassays, TaqMan diagnostic assays	Resistance to permethrin similar to other pyrethroids. Confirmed role of metabolic detoxification enzymes.	[165]
	Abamectin	Avermectin	8 populations from Northeast, Midwest, Southeast, and South Brazil	1.5 to 6.2 times	Detoxification enzymes (cytochrome CYP450s, GSTs)	Bioassays, enzyme activity assays	Resistance levels low, no major control failures observed, monitor long-term effectiveness	[166]
	Cartap	Nereistoxin derivative	8 populations from various Brazilian regions	1.5 to 6.4 times	Cytochrome CYP450s, GSTs involvement	Bioassays, enzyme activity assays	Populations from certain regions show resistance ratios that may lead to control failures	[166]
	Chlorfenapyr	Pyrrole	8 populations from different regions	1.4 to 4.6 times	CYP450 monooxygenase and GST activity	Bioassays, enzyme activity assays	Populations show low resistance, no cross-resistance to other insecticides like indoxacarb	[166]
	Indoxacarb	Oxadiazine	8 populations from various Brazilian region	1.1 to 3.3 times	No cross-resistance with metaflumizone	Bioassays, enzyme activity assays	No significant resistance was found; populations remain susceptible at recommended doses	[166]
	Metaflumizone	Semicarbazone	8 populations from different regions	2.5 to 21.2 times	Cross-resistance not observed	Bioassays, enzyme activity assays	Low to moderate resistance, some populations resistant at higher doses	[166]
	Spinosad	Spinosyn	IRA -Sel, IRA-Unsel, PLT-Sus	IRA-Sel: 48,900-fold, IRA-UnSel: 284-fold, PLT-Sus: 1-fold	Mutation G275E in nAChR α6 subunit	Bioassays, gene sequencing, TaqMan diagnostic assays	Resistance is linked to the G275E mutation in the nAChR α6 subunit. Resistance is autosomal, recessive, and monofactorial in the IRA-Sel strain.	[167]
	Cartap Hydrochloride	Nereistoxin derivative	GML2-Res, JDR1-Sus	GML2-Res: 537.1-fold, JDR1-Sus: 2.3-fold	Detoxification (hydrolases, GSTs, CYP450 monooxygenases)	Concentration-mortality bioassays, genetic studies	Cross-resistance with other insecticides (e.g., deltamethrin, methoxyfenozide). Synergism observed with inhibitors.	[168]
	Isocycloseram	Isoxazoline	JUA-2024, LGD-Clora, GVT-Aba,	GVT-Aba: 30.19-fold, JUA-2024: 1.06-fold, LGD-Clora: 1.06	Detoxification (cytochrome CYP450s)	Leaf-dip bioassay, LC_50_ and LC_99_ estimates	Early tolerance shifts, suggesting emerging resistance. Monitoring needed.	[169]
	Tolfenpyrad	Pyrazole	PTY-2024, PIE-2024, IRE-2023	PIE-2024: 2.15-fold, PTY-2024: 2.15-fold IRE-2023: 14.13-fold	Detoxification enzymes involved (esterases, GSTs)	Bioassay, diagnostic concentration estimation	Early signs of tolerance observed; need to manage emerging resistance	[169]
	Abamectin	Glutamate-gated chloride channel agonist	GVT-Aba, IRE-2024, SUM-2024	Up to 30-fold resistance	Esterase-based resistance	Synergism bioassays with S,S,S-tributyl phosphorotrithioate, and Piperonyl butoxide	Widespread resistance, linked to metabolic detoxification pathways	[169]
	Fipronil	Phenylpyrazole	Various populations	Resistance in multiple populations	GABA receptor antagonism	Diagnostic dose monitoring, synergism assays	Cross-resistance potential with other chloride channel modulators	[169]
Chile	Spinosad	Spinosyn	Azapa 1, Azapa 2, Lluta, Colín, Valdivia, S	Azapa 1: 1.83, Azapa 2: 2.04, Lluta: 2.32, Colín: 1.71, Valdivia: 1.41, S: 1	Increased MFO, EST, and GST activity	Bioassays with diagnostic concentration (1 mg/L), enzyme activity assays	Enhanced detoxification enzyme activity (MFO, EST) in resistant populations; possible cross-resistance with other insecticides	[170]
China	Tetraniliprole	Diamide insecticide	HL and 17 other populations	HL: 36.2-fold, other populations: 1.1–3.0-fold	CYP450 monooxygenase and GST activity	Leaf-dip bioassay, synergist tests (PBO, DEM, TPP), genetic analysis, enzyme activity assays	Tetraniliprole resistance in HL population is moderate; resistance inheritance is autosomal and polygenic	[171]
	Spinetoram	Spinosyn derivative	SPI-R (20th generation)	410.86-fold	Increased detoxification enzyme activities (CYP450s, GSTs, CarEs)	Bioassay (leaf dipping), synergist assays (PBO, DEM, TPP)	Resistance inheritance was polygenic, autosomal, incompletely recessive with fitness costs observed (extended larval stages, reduced adult longevity)	[172]
Greece	Chlorantraniliprole	Diamide insecticide	GR-IER-15-2	55-fold	G4903E, G4903V mutations	Genotyping, bioassays	G4903E and G4903V mutations linked to resistance; also carries I4746M mutation in low frequency	[164]
Spain	Chlorantraniliprole	Diamide insecticide	Ssus, Mur, Sres, Sus strains of *P. absoluta*	Ssus: 1, Mur: 18.2-fold, Sres: 44,614-fold, Sus: 1.7-fold	Overexpression of UGT34A23	RNA-seq, gene expression analysis, bioassays with transgenic *Drosophila melanogaster*	Resistance linked to metabolic detoxification (UGT overexpression), suggesting potential for synergists and molecular assays in management	[173]
	Chlorantraniliprole	Diamide insecticide	ES-MUR-14	8-fold	RyR mutations (low frequency of G4903E)	Bioassays, pyrosequencing	Moderate resistance, further research needed on potential other resistance mechanisms (e.g., detoxification)	[164]
Iran	Indoxacarb	Oxadiazine	Ar, Bu, Ya, Kr, Sh, Mo	Ar: 10.51-fold, Bu: 6.03-fold, Ya: 14.45-fold, Kr: 2.37-fold, Sh: 10.04-fold, Mo: 1.59-fold	Mutations in sodium channel (F1845Y and V1848I), detoxification (CYP450s, CarEs)	Bioassay, synergism assays with PBO, DEM, TPP, enzyme activity assays	Resistance primarily via target site mutations; no significant cross-resistance with other insecticides	[174]
Italy	Chlorantraniliprole, Flubendiamide	Diamide insecticides	IT-GELA-SD4, GR-Lab	>1000-fold in IT-GELA-SD4, less than 2-fold in GR-Lab	Ryanodine receptor mutations (G4903E, I4746M, G4903V)	Bioassays (leaf dip), pyrosequencing, radioligand binding studies	Resistance is linked to mutations in the RyR gene, autosomal and incompletely recessive inheritance; urgent resistance management strategies recommended	[164]

BR-GML1, Brazil–Gameleira (Bahia)–population 1–2014 (field strain); BR-PSQ, Brazil–Pesqueira (Pernambuco)–2014 (field strain); G4903E, Glycine at position 4903 replaced by Glutamic acid; I4746M, Isoleucine at position 4746 replaced by Methionine; L1014F, Leucine is replaced by Phenylalanine at position 1014; M918T, Methionine is replaced by Threonine at position 918; T929I, Threonine is replaced by Isoleucine at position 929; CYP450s, Cytochrome P450 monooxygenases; CarEs, Carboxylesterases; GST, glutathione S-transferase; IRA-Sel, Iraquara (BA, Brazil) spinosad-selected resistant strain; IRA-Unsel, Iraquara (BA, Brazil) population without insecticide selection; PLT-Sus, Pelotas (RS, Brazil) susceptible laboratory strain; G275E, Glycine at position 275 is replaced by Glutamic acid; nAChR α6 subunit, nicotinic acetylcholine receptor alpha-6 subunit; TaqMan diagnostic assay, Fluorescent probe-based real-time PCR tests using Taq DNA polymerase to detect specific genes or mutations; GML2-Res, João Dourado (BA, Brazil)–population 2–resistant strain; JDR1-Sus, João Dourado (BA, Brazil)–population 1–susceptible strain; JUA-2024, Juazeiro (BA, Brazil)–collected in 2024 (field population); LGD-Clora, Lagoa Grande (BA, Brazil)–chlorantraniliprole-selected/resistant strain; GVT-Aba, Gravatá (PE, Brazil)–abamectin-selected/resistant strain; LC_50_, Lethal concentration that kills 50% of the population; LC_99_, Lethal concentration that kills 99% of the population; PTY-2024, Paty do Alferes (RJ, Brazil)–collected in 2024 (field population); PIE-2024, Piedade (SP, Brazil)–collected in 2024 (field population); IRE-2023, Irecê (BA, Brazil)–collected in 2023 (field population); IRE-2024, Irecê (BA, Brazil)–collected in 2024 (field population); SUM-2024, Sumaré (SP, Brazil)–collected in 2024 (field population); GABA receptor, Gamma-aminobutyric acid receptor; Azapa 1, Azapa Valley–population 1 (field population); Azapa 2, Azapa Valley–population 2 (field population); Lluta, Lluta Valley field population; Colín, Colín location field population; Valdivia, Valdivia field population; S, Susceptible lab strain; MFO, Mixed-Function Oxidases; EST, Esterases; HL, Huailai; PBO, Piperonyl butoxide DEM, Diethyl maleate, TPP, Triphenyl phosphate; SPI-R, Spinetoram Resistant Strain; GR-IER-15-2 = Greece–Ierapetra (Kalogeri)–collected in 2015–population 2 (field strain); G4903V, Glycine at position 4903 replaced by Valine; RyR gene, Ryanodine Receptor gene; Ssus, Super susceptible strain; Mur, Moderately resistant strain; Sres, Strongly resistant strain; Sus, Susceptible strain; UGT34A23, uridine diphosphate-glycosyltransferase 34A23; RNA-seq, RNA sequencing; UGT, uridine di-phosphate-glycosyltransferase; ES-MUR-14, Spain–Murcia (Lorca)–collected in 2014 (field strain); Ar, Ardabil; Bu, Bushehr; Ya, Yazd; Kr, Kerman; Sh, ShahreKord; Mo, control; F1845Y, Phenylalanine at position 1845 is replaced by Tyrosine; V1848I, Valine at position 1848 is replaced by Isoleucine; IT-GELA-SD4, Italy–Gela population–selected for 4 cycles (chlorantraniliprole-selected strain); GR-Lab, Greece–laboratory-maintained susceptible strain.

**Table 4:** Pheromone trap types and their performance in monitoring and management of *Phthorimaea* (*Tuta*) *absoluta*.

Formulation	Active Pheromone Component(s)	Trap Type	Purpose	Reported Performance	Duration/Operational Note	Crop System	Country/Region	References
Delta traps with pheromones	(3E,8Z,11Z)-tetradecatrien-1-yl acetate and (3E,8Z)-tetradecadien-1-yl acetate	Delta trap	Monitoring population	Effective in capturing males to monitor *P. absoluta*	Replaced every 4 weeks	Greenhouse tomatoes	Albania	[231]
Pheromone lures	(3E,8Z,11Z)-tetradecatrien-1-yl acetate, (3E,8Z)-tetradecadien-1-yl acetate	Delta Trap	Monitoring Population	High attraction to *P. absoluta* moths in tomato and potato fields	Traps monitored daily for moth capture	Tomato & Potato	United States of America/Panama	[232]
Synthetic 0.8 mg pheromone	(3E,8Z,11Z)-tetradecatrien-1-yl acetate	Delta trap	Monitoring population	Captured significantly more males; highest capture rate observed in golden light traps	Replace pheromone every 4–6 weeks depending on temperature	Tomato	Pakistan	[233]
Tomato leaf miner lure	(Z)-11-hexadecenal and other similar component	Delta, Wota-T, Solar Light trap	Monitoring and mass trapping	Best performance in white-colored delta traps; significant differences observed with color and height placement	Delta traps changed every 5 weeks, Wota-T traps adjusted at 1–4 ft heights	Tomato	Nepal	[234]
Qlure-TAU^®^ Pheromone Capsule	(3E,8Z,11Z)-tetradecatrien-1-yl acetate	Plastic Container with Pheromone Capsule	Monitoring and Mass Trapping	White traps attracted the highest number of *P. absoluta* moths across all tested months	Traps serviced weekly; height fixed at 30 cm; color-specific analysis	Tomato	Egypt/Giza	[235]
Tomato leaf miner lure	(3E, 8Z, 11Z)-3,8,11-tetradecatrienyl acetate	Pan Water Traps	Monitoring male *P. absoluta*	No significant preference by color in most trials; green traps performed best	Pheromone lures replaced every 5–6 weeks, traps serviced every 1–2 weeks	Tomato	Tunisia	[236]
Synthetic sex pheromone capsule	(3E, 8Z, 11Z)-3,8,11-tetradecatrienyl acetate	Deltasan, Tutasan	Monitoring and mass trapping	Tutasan trap more effective during fruiting and ripening stages	Pheromone lures replaced weekly	Tomato	Côte d’Ivoire	[237]
*Tuta* Optima^®^ 0.8 mg	Synthetic sex pheromone for *T. absoluta*	Water pan trap, Palm weevil bucket trap, Sticky delta trap	Monitoring and mass trapping	Water pan trap most effective with 406 males/trap; green traps attracted the most males	Traps checked regularly; water traps used	Tomato	Egypt	[238]
Tomato leaf miner lure	(3E, 8Z, 11Z)-tetradecatrien-1-yl acetate, (3E, 8Z)-tetradecadien-1-yl acetate	Delta, Water Pan	Monitoring and Mass Trapping	Delta traps and water-filled bowls with pheromone traps effectively capture *P. absoluta*	Traps monitored weekly, pheromone replaced as per environmental conditions	Tomato, Solanaceous Crops	Global (Africa, Europe, Middle East, and Asia)	[239]
Tomato leaf miner lure	(3E,8Z,11Z)-tetradecatrien-1-yl acetate, (3E,8Z)-tetradecadien-1-yl acetate	Delta, Water Pan, Sticky Trap	Monitoring and Mass Trapping	Sticky traps significantly outperformed delta and water pan traps in capturing *P. absoluta* males	Traps serviced weekly (replacing pheromones and sticky sheets)	Tomato	United Arab Emirates	[106]
*P. absoluta* synthetic lure	(3E, 8Z, 11Z)-tetradecatrien-1-yl acetate, (3E, 8Z)-tetradecadien-1-yl acetate	Delta, Water pan trap	Monitoring and mass trapping	Effective in monitoring *P. absoluta* populations, achieving 35–70% reduction in pest density	Traps serviced regularly; lures replaced every 4–6 weeks	Tomato	Nigeria, Kenya, Benin	[240]
2.8 mg of pheromone compound mixture	(3E, 8Z, 11Z)-tetradecatrien-1-yl acetate	Wing Trap	Monitoring and Mass Trapping	The best trapping effect achieved at 0.5m height with ladder suspension method	Traps serviced regularly, lures replaced monthly, sticky boards every 3 days	Tomato	China/E-Shan	[241]

## Data Availability

No new data were created or analyzed in this study. Data sharing is not applicable to this article.

## References

[1] Aynalem B. (2022). Empirical Review of *Tuta absoluta* Meyrick Effect on the Tomato Production and Their Protection Attempts. Adv. Agric..

[2] Acharya R., Barman A.K., Sharma S.R., Kafle L., Kim S.-M., Lee K.-Y. (2023). Biology, distribution, and management of invasive South American tomato leafminer, *Tuta absoluta* (Meyrick) (Lepidoptera; Gelechiidae), in Asia. Arch. Insect Biochem. Physiol..

[3] Tonnang H.E.Z., Mohamed S.F., Khamis F., Ekesi S. (2015). Identification and Risk Assessment for Worldwide Invasion and Spread of *Tuta absoluta* with a Focus on Sub-Saharan Africa: Implications for Phytosanitary Measures and Management. PLoS ONE.

[4] Cocco A., Deliperi S., Lentini A., Mannu R., Delrio G. (2015). Seasonal phenology of *Tuta absoluta* (Lepidoptera: Gelechiidae) in protected and open-field crops under Mediterranean climatic conditions. Phytoparasitica.

[5] Balzan M.V., Moonen A.C. (2012). Management strategies for the control of *Tuta absoluta* (Lepidoptera: Gelechiidae) damage in open-field cultivations of processing tomato in Tuscany (Italy). EPPO Bull..

[6] Gharekhani G.H., Salek-Ebrahimi H. (2014). Evaluating the damage of *Tuta absoluta* (Meyrick) (Lepidoptera: Gelechiidae) on some cultivars of tomato under greenhouse condition. Arch. Phytopathol. Plant Prot..

[7] Pandey M., Bhattarai N., Pandey P., Chaudhary P., Katuwal D.R., Khanal D. (2023). A review on biology and possible management strategies of tomato leaf miner, *Tuta absoluta* (Meyrick), Lepidoptera: Gelechiidae in Nepal. Heliyon.

[8] Thrall P.H., Oakeshott J.G., Fitt G., Southerton S., Burdon J.J., Sheppard A., Russell R.J., Zalucki M., Heino M., Denison R.F. (2011). Evolution in agriculture: The application of evolutionary approaches to the management of biotic interactions in agro-ecosystems. Evol. Appl..

[9] Kim K.S., Sappington T.W. (2013). Population genetics strategies to characterize long-distance dispersal of insects. J. Asia-Pac. Entomol..

[10] Lee C.E. (2002). Evolutionary genetics of invasive species. Trends Ecol. Evol..

[11] Hauser M. (2011). A historic account of the invasion of Drosophila suzukii (Matsumura) (Diptera: Drosophilidae) in the continental United States, with remarks on their identification. Pest Manag. Sci..

[12] Kardum Hjort C., Paris J.R., Smith H.G., Dudaniec R.Y. (2024). Selection despite low genetic diversity and high gene flow in a rapid island invasion of the bumblebee, Bombus terrestris. Mol. Ecol..

[13] Arnó J., Gabarra R., Molina P., Godfrey K.E., Zalom F.G. (2019). *Tuta absoluta* (Lepidoptera: Gelechiidae) Success on Common Solanaceous Species from California Tomato Production Areas. Environ. Entomol..

[14] Cuthbertson A.G., Mathers J.J., Blackburn L.F., Korycinska A., Luo W., Jacobson R.J., Northing P. (2013). Population Development of *Tuta absoluta* (Meyrick) (Lepidoptera: Gelechiidae) under Simulated UK Glasshouse Conditions. Insects.

[15] Machekano H., Mutamiswa R., Nyamukondiwa C. (2018). Evidence of rapid spread and establishment of *Tuta absoluta* (Meyrick) (Lepidoptera: Gelechiidae) in semi-arid Botswana. Agric. Food Secur..

[16] Luo M., Huang L., Gui F., Judit A., Han P., Wan F., Zhang G., Huang C., Zhang Y. (2025). Rhythmic biosynthesis of sex pheromone modulates the calling and mating behaviors of *Tuta absoluta* (Lepidoptera: Gelechiidae). New Plant Prot..

[17] Gullino M.L., Albajes R., Al-Jboory I., Angelotti F., Chakraborty S., Garrett K.A., Hurley B.P., Juroszek P., Lopian R., Makkouk K. (2022). Climate Change and Pathways Used by Pests as Challenges to Plant Health in Agriculture and Forestry. Sustainability.

[18] Camargo R.A., Barbosa G.O., Possignolo I.P., Peres L.E., Lam E., Lima J.E., Figueira A., Marques-Souza H. (2016). RNA interference as a gene silencing tool to control *Tuta absoluta* in tomato (*Solanum lycopersicum*). PeerJ.

[19] Zhu J., Chen R., Liu J., Lin W., Liang J., Nauen R., Li S., Gao Y. (2024). Presence of Multiple Genetic Mutations Related to Insecticide Resistance in Chinese Field Samples of Two Phthorimaea Pest Species. Insects.

[20] Karanu S.W., Ajene I.J., Lelmen E.K., Ong’onge M.A., Akutse K.S., Khamis F.M. (2024). Biochemistry and transcriptomic analyses of *Phthorimaea absoluta* (Lepidoptera: Gelechiidae) response to insecticides. Sci. Rep..

[21] Page M.J., McKenzie J.E., Bossuyt P.M., Boutron I., Hoffmann T.C., Mulrow C.D., Shamseer L., Tetzlaff J.M., Akl E.A., Brennan S.E. (2021). The PRISMA 2020 statement: An updated guideline for reporting systematic reviews. BMJ.

[22] Tosevski I., Jovic J., Mitrovic M., Cvrkovic T., Krstic O., Krnjajic S. (2011). *Tuta absoluta* (Meyrick, 1917) (Lepidoptera, Gelechiidae): A new pest of tomato in Serbia. Pestic. I Fitomed..

[23] Guedes R.N.C., Picanço M.C. (2012). The tomato borer *Tuta absoluta* in South America: Pest status, management and insecticide resistance. EPPO Bull..

[24] Mm Filho V.E.F., Attygalle A.B., Meinwald J., Svatoš A., Jham G.N. (2000). Field trapping of tomato moth, *Tuta absoluta* with pheromone traps. J. Chem. Ecol..

[25] Desneux N., Wajnberg E., Wyckhuys K.A.G., Burgio G., Arpaia S., Narváez-Vasquez C.A., González-Cabrera J., Catalán Ruescas D., Tabone E., Frandon J. (2010). Biological invasion of European tomato crops by *Tuta absoluta*: Ecology, geographic expansion and prospects for biological control. J. Pest Sci..

[26] Tumuhaise V., Khamis F.M., Agona A., Sseruwu G., Mohamed S.A. (2016). First record of *Tuta absoluta* (Lepidoptera: Gelechiidae) in Uganda. Int. J. Trop. Insect Sci..

[27] Zekeya N., Ndakidemi P.A., Chacha M., Mbega E. (2017). Tomato Leafminer, *Tuta absoluta* (Meyrick 1917), an emerging agricultural pest in Sub-Saharan Africa: Current and prospective management strategies. Afr. J. Agric. Res..

[28] Zappalà L., Biondi A., Alma A., Al-Jboory I.J., Arnò J., Bayram A., Chailleux A., El-Arnaouty A., Gerling D., Guenaoui Y. (2013). Natural enemies of the South American moth, *Tuta absoluta*, in Europe, North Africa and Middle East, and their potential use in pest control strategies. J. Pest Sci..

[29] Campos M.R., Biondi A., Adiga A., Guedes R.N.C., Desneux N. (2017). From the Western Palaearctic region to beyond: *Tuta absoluta* 10 years after invading Europe. J. Pest Sci..

[30] Fand B.B., Shashank P.R., Suroshe S.S., Chandrashekar K., Meshram N.M., Timmanna H.N. (2020). Invasion risk of the South American tomato pinworm *Tuta absoluta* (Meyrick) (Lepidoptera: Gelechiidae) in India: Predictions based on MaxEnt ecological niche modelling. Int. J. Trop. Insect Sci..

[31] Guimapi R.Y.A., Mohamed S.A., Okeyo G.O., Ndjomatchoua F.T., Ekesi S., Tonnang H.E.Z. (2016). Modeling the risk of invasion and spread of *Tuta absoluta* in Africa. Ecol. Complex..

[32] Tarusikirwa V.L., Machekano H., Mutamiswa R., Chidawanyika F., Nyamukondiwa C. (2020). *Tuta absoluta* (Meyrick) (Lepidoptera: Gelechiidae) on the “Offensive” in Africa: Prospects for Integrated Management Initiatives. Insects.

[33] Mansour R., Brévault T., Chailleux A., Cherif A., Grissa-Lebdi K., Haddi K., Mohamed S.A., Nofemela R.S., Oke A., Sylla S. (2018). Occurrence, biology, natural enemies and management of *Tuta absoluta* in Africa. Entomol. Gen..

[34] Cherif A., Harbaoui K., Zappalà L., Grissa-Lebdi K. (2018). Efficacy of mass trapping and insecticides to control *Tuta absoluta* in Tunisia. J. Plant Dis. Prot..

[35] Adamou H., Adamou B., Garba M., Oumarou S., Gougari B., Abou M., Kimba A., Delmas P. (2016). Confirmation of the presence of *Tuta absoluta* (meyrick)(lepidoptera: Gelechiidae) in Niger (West Africa). Int. J. Sci. Environ. Technol..

[36] Pfeiffer D.G., Muniappan R., Sall D., Diatta P., Diongue A., Dieng E.O. (2013). First record of *Tuta absoluta* (lepidoptera: Gelechiidae) in Senegal. Fla. Entomol..

[37] Mahmoud M.E.E., Mohammed E.S., Mohamed S.A., Khamis F.M., Ekesi S. (2020). Development and implementation of a sustainable IPM and surveillance program for the invasive tomato leafminer, Tuta Absoluta (Meyrick) in Sudan. Athens J. Sci..

[38] Retta A.N., Berhe D.H. (2015). Tomato leaf miner–*Tuta absoluta* (Meyrick), a devastating pest of tomatoes in the highlands of Northern Ethiopia: A call for attention and action. Res. J. Agric. Environ. Manag..

[39] Kinyanjui G., Khamis F.M., Ombura F.L.O., Kenya E.U., Ekesi S., Mohamed S.A. (2021). Distribution, abundance and natural enemies of the invasive tomato leafminer, *Tuta absoluta* (Meyrick) in Kenya. Bull. Entomol. Res..

[40] Chidege M., Al-zaidi S., Hassan N., Julie A., Kaaya E., Mrogoro S. (2016). First record of tomato leaf miner *Tuta absoluta* (Meyrick) (Lepidoptera: Gelechiidae) in Tanzania. Agric. Food Secur..

[41] Abass M.S., Msiska K.K., Chomba M.D., Mudenda M., Mukuwa P.S.C. (2019). First Report of *Tuta absoluta* (Meyrick) in Zambia. Afr. Phytosanitary J..

[42] Bajracharya A.S.R., Mainali R.P., Bhat B., Bista S., Shashank P.R., Meshram N.M. (2016). The first record of South American tomato leaf miner, *Tuta absoluta* (Meyrick 1917) (Lepidoptera: Gelechiidae) in Nepal. J. Entomol. Zool. Stud..

[43] Jason D.S., Thomas D., Raphael M., Efrem-Fred N., Steven T., Ramasamy S. (2018). Host Range of the Invasive Tomato Pest *Tuta absoluta* Meyrick (Lepidoptera: Gelechiidae) on Solanaceous Crops and Weeds in Tanzania. Fla. Entomol..

[44] Mahlangu L., Sibisi P., Nofemela R.S., Ngmenzuma T., Ntushelo K. (2022). The Differential Effects of *Tuta absoluta* Infestations on the Physiological Processes and Growth of Tomato, Potato, and Eggplant. Insects.

[45] Terzidis A.N., Wilcockson S., Leifert C. (2014). The tomato leaf miner (*Tuta absoluta*): Conventional pest problem, organic management solutions?. Org. Agric..

[46] Vivekanandhan P., Swathy K., Sarayut P., Patcharin K. (2024). Biology, classification, and entomopathogen-based management and their mode of action on *Tuta absoluta* (Meyrick) in Asia. Front. Microbiol..

[47] Mathi M.C. (2024). Insect Pests: Identification, Behaviour and Management. Field to Plenty: Exploring the World of General Agriculture.

[48] Corro P., Metz M. (2021). Classification of *Tuta absoluta* (Meyrick, 1917) (Lepidoptera: Gelechiidae: Gelechiinae: Gnorimoschemini) Based on Cladistic Analysis of Morphology. Proc. Entomol. Soc. Wash..

[49] Bettaibi A., Mezghani Khemakhem M., Bouktila D., Makni H., Makni M. (2012). Genetic Variability of the Tomato Leaf Miner (*Tuta absoluta* Meirick; Lepidoptera: Gelechiidae), in Tunisia, Inferred from RAPD-PCR. Chil. J. Agric. Res..

[50] Ghedir A., Oueslati N., Gasmi L., Khorramnejad A., Said K., Ometto L. (2024). Genetic diversity in the tomato leafminer *Tuta absoluta* (Meyrick) in Tunisia. Phytoparasitica.

[51] Li X.-w., Fu K.-y., Guo W.-c., Wang T.-z., Lu Y.-b. (2022). The complete mitochondrial genome of *Tuta absoluta* (Lepidoptera: Gelechiidae) and genetic variation in two newly invaded populations in China. J. Asia-Pac. Entomol..

[52] Mehrkhou F., Güz N., Korkmaz E., Çağatay N.S. (2021). Analysis of genetic variation in an important pest, *Tuta absoluta*, and its microbiota witha new bacterial endosymbiont. Turk. J. Agric. For..

[53] Javal M., Ndiaye A., Loiseau A., Bocar B.A., Garba M., Brévault T., Gauthier N. (2025). *Tuta absoluta*’s population genetic structure across Africa: Two well-delineated but weakly differentiated groups suggesting few introductions and significant gene flow. Agric. For. Entomol..

[54] Wang Y., Tian X., Wang H., Castañé C., Arnó J., Wu S., Xian X., Desneux N., Liu W., Zhang G. (2023). Genetic diversity and genetic differentiation pattern of *Tuta absoluta* across China. Entomol. Gen..

[55] Zibaee I., Mahmood K., Esmaeily M., Bandani A.R., Kristensen M. (2018). Organophosphate and pyrethroid resistances in the tomato leaf miner *Tuta absoluta* (Lepidoptera: Gelechiidae) from Iran. J. Appl. Entomol..

[56] Haddi K., Berger M., Bielza P., Cifuentes D., Field L.M., Gorman K., Rapisarda C., Williamson M.S., Bass C. (2012). Identification of mutations associated with pyrethroid resistance in the voltage-gated sodium channel of the tomato leaf miner (*Tuta absoluta*). Insect Biochem. Mol. Biol..

[57] Haddi K., Berger M., Bielza P., Rapisarda C., Williamson M.S., Moores G., Bass C. (2017). Mutation in the ace-1 gene of the tomato leaf miner (*Tuta absoluta*) associated with organophosphates resistance. J. Appl. Entomol..

[58] Yalcin M., Mermer S., Kozaci L.D., Turgut C. (2015). Insecticide resistance in two populations of *Tuta absoluta* (Meyrick, 1917)(Lepidoptera: Gelechiidae) from Turkey. Turk. J. Entomol..

[59] Azizi M., Khajehali J. (2022). Evaluation of resistance to abamectin in the populations of *Tuta absoluta* (Lepidoptera: Gelechiidae), collected from Isfahan Province, Iran. J. Agric. Sci. Technol..

[60] Lewald K.M., Tabuloc C.A., Godfrey K.E., Arnó J., Perini C.R., Guedes J.C., Chiu J.C. (2023). Genome Assembly and Population Sequencing Reveal Three Populations and Signatures of Insecticide Resistance of *Tuta absoluta* in Latin America. Genome Biol. Evol..

[61] Cesari M., Maistrello L., Ganzerli F., Dioli P., Rebecchi L., Guidetti R. (2014). A pest alien invasion in progress: Potential pathways of origin of the brown marmorated stink bug Halyomorpha halys populations in Italy. J. Pest Sci..

[62] Roques A., Auger-Rozenberg M.A., Blackburn T., Garnas J., Pyšek P., Rabitsch W., Richardson D., Wingfield M., Liebhold A., Duncan R. (2016). Temporal and interspecific variation in rates of spread for insect species invading Europe during the last 200 years. Biol. Invasions.

[63] Guillemaud T., Blin A., Le Goff I., Desneux N., Reyes M., Tabone E., Tsagkarakou A., Niño L., Lombaert E. (2015). The tomato borer, *Tuta absoluta*, invading the Mediterranean Basin, originates from a single introduction from Central Chile. Sci. Rep..

[64] Vivekanandhan P., Swathy K., Siripan T., Sarayut P., Patcharin K. (2024). First report of Solanum indicum as a new host of *Tuta absoluta* (Lepidoptera: Gelechiidae). J. Integr. Pest Manag..

[65] Zhang G.-f., Ma D.-y., Wang Y.-s., Gao Y.-h., Liu W.-x., Zhang R., Fu W.-j., Xian X.-q., Wang J., Kuang M. (2020). First report of the South American tomato leafminer, *Tuta absoluta* (Meyrick), in China. J. Integr. Agric..

[66] Gedefaw Y., Fekadu A., Hinsermu M. (2024). Identification and Management of Major Diseases and Arthropod Pests of Tomato in Ethiopia: A Technical Manual.

[67] Rasheed V.A., Rao S.R.K., Babu T.R., Krishna M.T., Reddy B.V.B., Naidu G.M. (2018). Biology and morphometrics of tomato pinworm, *Tuta absoluta* (Meyrick) on tomato. Int. J. Curr. Microbiol. Appl. Sci.

[68] Ayalew G., Tuke B., Asfaw T. (2014). Disposing of Fruit to Manage Tuta absoluta on Tomato.

[69] Abdel Farag El-Shafie H., Abdel Farag El-Shafie H. (2020). *Tuta absoluta* (Meyrick) (Lepidoptera: Gelechiidae): An Invasive Insect Pest Threatening the World Tomato Production. Invasive Species—Introduction Pathways, Economic Impact, and Possible Management Options.

[70] Guenaoui Y. (2008). Nouveau ravageur de la tomate en Algérie: Première observation de *Tuta absoluta*, mineuse de la tomate invasive, dans la région de Mostaganem, au printemps 2008: Végétaux du soleil. Phytoma La Défense Des Végétaux.

[71] EPPO (2008). EPPO Reporting Service n. 1, 9.

[72] EPPO (2010). EPPO Reporting Service n. 1, 2, 3, 6, 8.

[73] Seplyarsky V., Weiss M., Haberman A. (2010). *Tuta absoluta* Povolny (Lepidoptera: Gelechiidae), a new invasive species in Israel. Phytoparasitica.

[74] Han P., Bayram Y., Shaltiel-Harpaz L., Sohrabi F., Saji A., Esenali U.T., Jalilov A., Ali A., Shashank P.R., Ismoilov K. (2019). *Tuta absoluta* continues to disperse in Asia: Damage, ongoing management and future challenges. J. Pest Sci..

[75] Saidov N., Ramasamy S., Mavlyanova R., Qurbonov Z. (2018). First Report of Invasive South American Tomato Leaf Miner *Tuta absoluta* (Meyrick) (Lepidoptera: Gelechiidae) in Tajikistan. Fla. Entomol..

[76] Urbaneja A., Vercher R., Navarro-Llopis V., Porcuna Coto J.L., García-Marí F. (2007). La polilla del tomate, ‘*Tuta absoluta*’. Phytoma Esp. La Rev. Prof. De Sanid. Veg..

[77] EPPO (2009). EPPO Reporting Service n. 1, 2, 3, 6, 8, 9, 10, 11.

[78] Viggiani G., Filella F., Delrio G., Ramassini W., Foxi C. (2009). *Tuta absoluta*, nuovo lepidottero segnalato in Italia. L’inform. Agrar..

[79] Roditakis E., Papachristos D., Roditakis N.E. (2010). Current status of the tomato leafminer *Tuta absoluta* in Greece. EPPO Bull..

[80] Izhevsky S.S., Akhatov A.K., Sinyov S.Y. (2011). *Tuta absoluta* has been detected in Russia. Zashchita I Karantin Rasteniĭ.

[81] EPPO (2011). EPPO Reporting Service n. 4, 11.

[82] Sadique M., Ishtiaq M., Naeem-Ullah U., Faried N. (2022). Spatio-temporal distribution of *Tuta absoluta* (Meyrick 1917) (Lepidoptera: Gelechiidae) from Pakistan. Int. J. Trop. Insect Sci..

[83] IPCC (2014). Intergovernmental Panel on Climate Change, Climate change 2014: Impacts, Adaptation, and Vulnerability. Part A: Global and sectoral aspects. Contribution of Working Group II to the Fifth Assessment Report of the Intergovernmental Panel on Climate Change.

[84] Konan K.A.J., Ouali-Ngoran S.W.M., Fondio L., Ochou G.O., Koné D., Desneux N., Martin T. (2022). Geographical distribution and host range status of *Tuta absoluta* Meyrick (Lepidoptera: Gelechiidae) in Côte d’Ivoire. Int. J. Trop. Insect Sci..

[85] Dangles O., Carpio C., Barragan A.R., Zeddam J.L., Silvain J.F. (2008). Temperature as a key driver of ecological sorting among invasive pest species in the tropical andes. Ecol. Appl..

[86] Xu B., Kong D., Zhang G., Huang C., Zhang G., Wan F. (2025). Genome-wide Characterization of Heat Shock Protein (HSP) Genes in *Tuta (Phthorimaea) Absoluta*: Insights into Thermal Stress Response. Neotrop. Entomol..

[87] Zannou A.J., Karaca M.M., Karut K. (2024). Effect of constant and fluctuating low temperature on the survival of *Tuta absoluta* pupae. Bull. Entomol. Res..

[88] Bacci L., da Silva É.M., Martins J.C., Soares M.A., Campos M.R.d., Picanço M.C. (2019). Seasonal variation in natural mortality factors of *Tuta absoluta* (Lepidoptera: Gelechiidae) in open-field tomato cultivation. J. Appl. Entomol..

[89] Johansson B.G., Jones T.M. (2007). The role of chemical communication in mate choice. Biol. Rev..

[90] Römer H. (2020). Insect acoustic communication: The role of transmission channel and the sensory system and brain of receivers. Funct. Ecol..

[91] Ombuya A., Guo J., Liu W. (2025). Insect Mating Behaviors: A Review of the Regulatory Role of Neuropeptides. Insects.

[92] Ou X., Li X., Xu B., Wang Y., Zhang G., Liu W., Wan F., Jiang H., Haddi K., Huang C. (2025). Expression and sex pheromone-binding characteristics of pheromone-binding protein 3 in *Tuta absoluta* (Lepidoptera: Gelechiidae). Pestic. Biochem. Physiol..

[93] Rana A., Sharma D., Choudhary K., Kumari P., Ruchika K., Yangchan J., Kumar S. (2024). Insight into insect odorant binding proteins: An alternative approach for pest management. J. Nat. Pestic. Res..

[94] Fan J., Francis F., Liu Y., Chen J.L., Cheng D.F. (2011). An overview of odorant-binding protein functions in insect peripheral olfactory reception. Genet. Mol. Res. GMR.

[95] Qu C., Yan J., Yan Z., Li R., Liu Y., Lin A., Fu Y., Luo C., Kang Z., Wang R. (2025). TabsPBP2, a Pheromone-Binding Protein Highly Expressed in Male Antennae of *Tuta absoluta*, Binds Sex Pheromones and Tomato Volatiles. Biomolecules.

[96] Deisig N., Dupuy F., Anton S., Renou M. (2014). Responses to Pheromones in a Complex Odor World: Sensory Processing and Behavior. Insects.

[97] Domínguez A., López S., Bernabé A., Guerrero Á., Quero C. (2019). Influence of Age, Host Plant and Mating Status in Pheromone Production and New Insights on Perception Plasticity in *Tuta Absoluta*. Insects.

[98] Gonçalves G.A.d.S., Haddi K., Ribas N.d.S., Santos K.C.P., Tschoeke L.F.P., Lima E. (2024). Age, weight, and mating status of the male influence female choice and reproductive success in *Tuta absoluta*. Entomol. Gen..

[99] Lee M.S., Albajes R., Eizaguirre M. (2014). Mating behaviour of female *Tuta absoluta* (Lepidoptera: Gelechiidae): Polyandry increases reproductive output. J. Pest Sci..

[100] Lewis S., South A., Burns R., Al-Wathiqui N. (2011). Nuptial gifts. Curr. Biol..

[101] Wedell N., Tregenza T., Simmons L.W. (2008). Nuptial gifts fail to resolve a sexual conflict in an insect. BMC Evol. Biol..

[102] South A., Lewis S. (2012). Effects of male ejaculate on female reproductive output and longevity in Photinus fireflies. Can. J. Zool..

[103] Liu W., Xu J., Zhang R. (2016). The optimal sex pheromone release rate for trapping the codling moth *Cydia pomonella* (Lepidoptera: Tortricidae) in the field. Sci. Rep..

[104] Chermiti B., Abbes K. (2012). Comparison of pheromone lures used in mass trapping to control the tomato leafminer *Tuta absoluta* (Meyrick, 1917) in industrial tomato crops in Kairouan (Tunisia). EPPO Bull..

[105] Sadique M., Ishtiaq M., Qayyum M.A., Alkherb W.A.H., Abbasi A., Arshad M., Ullah U.N., Faried N., Akram M.I., Rebouh N.Y. (2025). Comparison of Pheromone Lures and Sticky Pad Color for Capturing *Tuta absoluta* (Lepidoptera: Gelechiidae). Insects.

[106] Sabbahi R., Azzaoui K. (2022). The effectiveness of pheromone traps in controlling the tomato leafminer, *Tuta absoluta*, in the United Arab Emirates. J. Plant Dis. Prot..

[107] Komal J., Desai H.R., Samal I., Mastinu A., Patel R.D., Kumar P.V.D., Majhi P.K., Mahanta D.K., Bhoi T.K. (2023). Unveiling the Genetic Symphony: Harnessing CRISPR-Cas Genome Editing for Effective Insect Pest Management. Plants.

[108] Faber N.R., Ashok K., Venkatesan T., Wertheim B., Bulgarella M. (2026). Leveraging advances in RNAi and CRISPR for improved biological pest control. Curr. Opin. Insect Sci..

[109] Hough J., Howard J.D., Brown S., Portwood D.E., Kilby P.M., Dickman M.J. (2022). Strategies for the production of dsRNA biocontrols as alternatives to chemical pesticides. Front. Bioeng. Biotechnol..

[110] Singh S., Rahangdale S., Pandita S., Saxena G., Upadhyay S.K., Mishra G., Verma P.C. (2022). CRISPR/Cas9 for Insect Pests Management: A Comprehensive Review of Advances and Applications. Agriculture.

[111] Zhao Y., Li L., Wei L., Wang Y., Han Z. (2024). Advancements and Future Prospects of CRISPR-Cas-Based Population Replacement Strategies in Insect Pest Management. Insects.

[112] Guerra F., De Rouck S., Verhulst E.C. (2026). SYNCAS-mediated CRISPR-Cas9 genome editing in the Jewel wasp, Nasonia vitripennis. Insect Mol. Biol..

[113] Bouyer J., Vreysen M.J.B. (2019). Concerns about the feasibility of using “precision guided sterile males” to control insects. Nat. Commun..

[114] Kandul N.P., Liu J., Sanchez C.H.M., Wu S.L., Marshall J.M., Akbari O.S. (2019). Transforming insect population control with precision guided sterile males with demonstration in flies. Nat. Commun..

[115] Aslam H.B., Amin M.R., Rahman M.M. (2026). CRISPR/CAS9 mediated genome editing in insects: Application in functional genomics and pest management. Discov. Agric..

[116] Wang Y., Zhang C., Li M.J., Iqbal A., Ahmed K.S., Idrees A., Habiba, Yang B.M., Jiang L. (2025). Exploring the Role of Pheromones and CRISPR/Cas9 in the Behavioral and Olfactory Mechanisms of Spodoptera frugiperda. Insects.

[117] El-Awaad E., Merzendorfer H. (2025). CRISPR/Cas: An emerging molecular technology for biological control of fall armyworm. New Plant Prot..

[118] Saini A., Sharma N., Sharma N., Kumari N., Sharma M., Singh B., Thakur A.K. (2026). Precision pest management: Genome editing tools, specifically CRISPR/Cas9 and future prospects. Pestic. Biochem. Physiol..

[119] Guo C., Ma X., Gao F., Guo Y. (2023). Off-target effects in CRISPR/Cas9 gene editing. Front. Bioeng. Biotechnol..

[120] Movahedi A., Aghaei-Dargiri S., Li H., Zhuge Q., Sun W. (2023). CRISPR Variants for Gene Editing in Plants: Biosafety Risks and Future Directions. Int. J. Mol. Sci..

[121] Archives J., Blog G.M.J. (2025). Host Plant Suitability of *Tuta absoluta* Meyrick (Lepidoptera: Gelechiidae) on Four Plant Species. J. Environ. Agric. Sci. (JEAS) Erdogan.

[122] Abdullah A., Ullah M.I., Raza A.B.M., Arshad M., Afzal M. (2019). Host plant selection affects biological parameters in armyworm, Spodoptera litura (Lepidoptera: Noctuidae). Pak. J. Zool..

[123] Negi S., Sharma P., Sharma K., Verma S. (2018). Effect of host plants on developmental and population parameters of invasive leafminer, *Tuta absoluta* (Meyrick) (Lepidoptera: Gelechiidae). Phytoparasitica.

[124] Li X., Cai X., Shang L., Wang Y., Haq I.U., Wang J., Hou Y. (2025). Adaptability Analysis of *Tuta absoluta* to Different Hosts and Related Salivary Genes Identification. J. Agric. Food Chem..

[125] Desneux N., Luna M.G., Guillemaud T., Urbaneja A. (2011). The invasive South American tomato pinworm, *Tuta absoluta*, continues to spread in Afro-Eurasia and beyond: The new threat to tomato world production. J. Pest Sci..

[126] Salama H.S., Shehata I.E.-S., Ebada I.M., Fouda M., Ismail I.A.E.-K. (2019). Prediction of annual generations of the tomato leaf miner *Tuta absoluta* on tomato cultivations in Egypt. Bull. Natl. Res. Cent..

[127] Saha A., Tripathy S. (2025). Metamorphosis in insects—The inherent mechanism of survival. Agric. J. World.

[128] Loyani L., Bradshaw K., Machuve D. (2021). Segmentation of *Tuta Absoluta* ’s Damage on Tomato Plants: A Computer Vision Approach. Appl. Artif. Intell..

[129] Rakesh V., Karthik R., Chandana C.R., Jayanth B.V., Ajith S. (2026). Unravelling the role of simulation modelling techniques (SMTs) in insect pest management. Pest Manag. Sci..

[130] Mengasha G.G., Salo K.S., Kassaye A., Terefe H., Fentahun Z., Suure M.T., Atumo T.T. (2026). Spatiotemporal dynamics and drivers of *Phthorimaea absoluta* infestation in tomatoes: Inferences for identifying measures to enhance components of integrated pest management strategies. Crop Prot..

[131] Rossini L., Severini M., Contarini M., Speranza S. (2019). A novel modelling approach to describe an insect life cycle vis-à-vis plant protection: Description and application in the case study of *Tuta absoluta*. Ecol. Model..

[132] Neta A., Levi Y., Morin E., Morin S. (2023). Seasonal forecasting of pest population dynamics based on downscaled SEAS5 forecasts. Ecol. Model..

[133] Bacci L., da Silva É.M., Martins J.C., da Silva R.S., Chediak M., Milagres C.C., Picanço M.C. (2021). The seasonal dynamic of *Tuta absoluta* in Solanum lycopersicon cultivation: Contributions of climate, plant phenology, and insecticide spraying. Pest Manag. Sci..

[134] Salama S.S., Ammar M., Gomaa E.E.L.S., Ali S.S., Abass A.A.E. (2023). Effect of certain environmental factors on population dynamics of *Tuta absoluta* (Meyrick) in tomato plant (*Solanum lycopersicum*) at Giza Governorate. Int. J. Theor. Appl. Res..

[135] Rupawate P.S., Roylawar P., Khandagale K., Gawande S., Ade A.B., Jaiswal D.K., Borgave S. (2023). Role of gut symbionts of insect pests: A novel target for insect-pest control. Front. Microbiol..

[136] Lv C., Huang Y.-Z., Luan J.-B. (2024). Insect-microbe symbiosis-based strategies offer a new avenue for the management of insect pests and their transmitted pathogens. Crop Health.

[137] Liu X.-D., Guo H.-F. (2019). Importance of endosymbionts Wolbachia and Rickettsia in insect resistance development. Curr. Opin. Insect Sci..

[138] Mallika A.R., Rajasri M. (2025). Endosymbiont-based strategies in insect pest control: Innovations and applications. Int. J. Adv. Biochem. Res..

[139] Ratzka C., Gross R., Feldhaar H. (2012). Endosymbiont Tolerance and Control within Insect Hosts. Insects.

[140] Gao H., Yin X.-J., Fan Z.-H., Gu X.-H., Su Z.-Q., Luo B.-R., Qiu B.-L., Zhang L.-H. (2025). Effects of Endosymbionts on the Nutritional Physiology and Biological Characteristics of Whitefly Bemisia tabaci. Insects.

[141] Panigrahi C.K., Paul M., Verma A., Satapathy S.N., Adhikari B., Panigrahi S., Mohapatra P., Devi B.R., Choudhary A., Gawaria J. (2025). Endosymbionts in Insects: Functional Roles and Applications in Pest Management. South Asian J. Parasitol..

[142] Sahani S., Saha T., Kumari K., Ansar M. (2023). Diversity of bacterial communities associated with the gut of the fall armyworm, *Spodoptera frugiperda* (J.E. Smith) (Lepidoptera: Noctuidae) in Eastern India. Phytoparasitica.

[143] Paniagua Voirol L.R., Frago E., Kaltenpoth M., Hilker M., Fatouros N.E. (2018). Bacterial Symbionts in Lepidoptera: Their Diversity, Transmission, and Impact on the Host. Front. Microbiol..

[144] Zare Banadkuki S., Rahmani S., Bandani A.R. (2024). Bacterial communities of two populations of *Tuta absoluta* (Meyrick) (Lepidoptera: Gelechiidae). J. Asia-Pac. Entomol..

[145] Shang L.-H., Cai X.-Y., Li X.-J., Wang Y.-Z., Wang J.-D., Hou Y.-M. (2024). Role of Gut Bacteria in Enhancing Host Adaptation of *Tuta absoluta* to Different Host Plants. Insects.

[146] Li J., Yu Y., Zulu L., Xu N., Pan Y., He W., Liu X., Rao Q. (2026). Gut Symbiont-Driven Adaptive Evolution of Herbivorous Insect–Plant Interactions and Its Ecological Implications. Plants.

[147] Chamani M., Dadpour M., Dehghanian Z., Panahirad S., Chenari Bouket A., Oszako T., Kumar S. (2025). From Digestion to Detoxification: Exploring Plant Metabolite Impacts on Insect Enzyme Systems for Enhanced Pest Control. Insects.

[148] Godavari A.K., Kumar H.K., Bharathi M.C. (2024). Endosymbionts mediated detoxification of insecticides in insects. Int. J. Adv. Biochem. Res..

[149] Elston K.M., Leonard S.P., Geng P., Bialik S.B., Robinson E., Barrick J.E. (2022). Engineering insects from the endosymbiont out. Trends Microbiol..

[150] Azrag A.G.A., Obala F., Tonnang H.E.Z., Hogg B.N., Ndlela S., Mohamed S.A. (2023). Predicting the impact of climate change on the potential distribution of the invasive tomato pinworm *Phthorimaea absoluta* (Meyrick) (Lepidoptera: Gelechiidae). Sci. Rep..

[151] Gao T., Feng R., Liu Z., Zhu Z. (2025). Modeling the Potential Climatic Suitability and Expansion Risk of *Tuta absoluta* (Meyrick, 1917) (Lepidoptera: Gelechiidae) Under Future Climate Scenarios. Insects.

[152] Zhou J., Luo W., Song S., Wang Z., Zhu X., Gao S., He W., Xu J. (2024). The Impact of High-Temperature Stress on the Growth and Development of *Tuta absoluta* (Meyrick). Insects.

[153] Guimapi R.A., Srinivasan R., Tonnang H.E., Sotelo-Cardona P., Mohamed S.A. (2020). Exploring the Mechanisms of the Spatiotemporal Invasion of *Tuta absoluta* in Asia. Agriculture.

[154] Xue Y., Huang C., Xian X., Liu W., Wan F., Desneux N., Zhang G., Zhang Y. (2025). Modeling the spread patterns and climatic niche dynamics of the tomato leaf miner *Tuta absoluta* following its invasion of China. J. Pest Sci..

[155] Saher R., Middel A., Stephen H., Ahmad S. (2022). Assessing the Microclimate Effects and Irrigation Water Requirements of Mesic, Oasis, and Xeric Landscapes. Hydrology.

[156] Silva G.A., Picanço M.C., Bacci L., Crespo A.L.B., Rosado J.F., Guedes R.N.C. (2011). Control failure likelihood and spatial dependence of insecticide resistance in the tomato pinworm, *Tuta absoluta*. Pest Manag. Sci..

[157] Ong’onge M.A., Ajene I.J., Runo S., Sokame B.M., Khamis F.M. (2023). Population dynamics and insecticide resistance in *Tuta absoluta* (Lepidoptera: Gelechiidae), an invasive pest on tomato in Kenya. Heliyon.

[158] Kim J.I., Joo Y.R., Kwon M., Kim G.H., Lee S.H. (2012). Mutation in ace1 associated with an insecticide resistant population of Plutella xylostella. J. Asia-Pac. Entomol..

[159] Ye X., Yang L., Stanley D., Li F., Fang Q. (2017). Two Bombyx mori acetylcholinesterase genes influence motor control and development in different ways. Sci. Rep..

[160] Zhang F., Mu M., Wang Z., Zhang H., Song Y., Xiao R. (2025). The Characteristics and Functions of SSRs and SNPs Based on the Transcriptome of *Tuta absoluta* Exposed to Different Concentrations of Abamectin and Chlorantraniliprole. Insects.

[161] Ma X., Qu C., Yao J., Xia J., Luo C., Guedes R.N.C., Wang R. (2024). Resistance monitoring of diamide insecticides and characterization of field-evolved chlorantraniliprole resistance among Chinese populations of the tomato pinworm Phthorimaea (=*Tuta*) *absoluta* (Lepidoptera: Gelechiidae). Pestic. Biochem. Physiol..

[162] Zhang Y.-b., Li H., Han P., Tian X.-c., Wang H., Geng L.-l., Zhang J., Liu W.-x., Wan F.-h., Guedes R.-N. (2025). Monitoring the insecticide susceptibility of a newly introduced invasive species, *Tuta absoluta* (Meyrick), in China. Crop Prot..

[163] Guedes R.N.C., Roditakis E., Campos M.R., Haddi K., Bielza P., Siqueira H.A.A., Tsagkarakou A., Vontas J., Nauen R. (2019). Insecticide resistance in the tomato pinworm *Tuta absoluta*: Patterns, spread, mechanisms, management and outlook. J. Pest Sci..

[164] Roditakis E., Steinbach D., Moritz G., Vasakis E., Stavrakaki M., Ilias A., García-Vidal L., Martínez-Aguirre M.d.R., Bielza P., Morou E. (2017). Ryanodine receptor point mutations confer diamide insecticide resistance in tomato leafminer, *Tuta absoluta* (Lepidoptera: Gelechiidae). Insect Biochem. Mol. Biol..

[165] Silva W.M., Berger M., Bass C., Balbino V.Q., Amaral M.H., Campos M.R., Siqueira H.A. (2015). Status of pyrethroid resistance and mechanisms in Brazilian populations of *Tuta absoluta*. Pestic. Biochem. Physiol..

[166] Silva T.B.M., Silva W.M., Campos M.R., Silva J.E., Ribeiro L.M.S., Siqueira H.A.A. (2016). Susceptibility levels of *Tuta absoluta* (Meyrick) (Lepidoptera: Gelechiidae) to minor classes of insecticides in Brazil. Crop Prot..

[167] Silva W.M., Berger M., Bass C., Williamson M., Moura D.M.N., Ribeiro L.M.S., Siqueira H.A.A. (2016). Mutation (G275E) of the nicotinic acetylcholine receptor α6 subunit is associated with high levels of resistance to spinosyns in *Tuta absoluta* (Meyrick) (Lepidoptera: Gelechiidae). Pestic. Biochem. Physiol..

[168] Pereira D.L., Silva P.A.F., Langa T.P., de Oliveira M., Ribeiro L.M.S., Siqueira H.A.A. (2023). Recent assessment and characterization of *Tuta absoluta* resistance to cartap hydrochloride. Pestic. Biochem. Physiol..

[169] Quintela V., Langa T.P., Dantas J.V.C.R., de Oliveira M., Siqueira H.A.A. (2026). Susceptibility of *Phthorimaea absoluta* (Meyrick) (Lepidoptera: Gelechiidae) to novel and established insecticides in Brazil: Resistance survey, baseline, and implications for management. Pest Manag. Sci..

[170] Reyes M., Rocha K., Alarcón L., Siegwart M., Sauphanor B. (2012). Metabolic mechanisms involved in the resistance of field populations of *Tuta absoluta* (Meyrick) (Lepidoptera: Gelechiidae) to spinosad. Pestic. Biochem. Physiol..

[171] Qu C., Yao J., Huang J., Che W., Fang Y., Luo C., Wang R. (2024). Tetraniliprole resistance in field-collected populations of *Tuta absoluta* (Lepidoptera: Gelechiidae) from China: Baseline susceptibility, cross-resistance, inheritance, and biochemical mechanism. Pestic. Biochem. Physiol..

[172] Xie J., Turak R., Liu N., Zhuang Z., Liu X., Song Y. (2025). Spinetoram resistance in *Tuta absoluta*: Selection, inheritance, biochemical mechanism and fitness cost. Pestic. Biochem. Physiol..

[173] Grant C., Singh K.S., Hayward A., Hunt B.J., Troczka B.J., Pym A., Ahn S.-J., Zeng B., Gao C.-F., Leroux A. (2023). Overexpression of the UDP-glycosyltransferase UGT34A23 confers resistance to the diamide insecticide chlorantraniliprole in the tomato leafminer, *Tuta absoluta*. Insect Biochem. Mol. Biol..

[174] Aboutalebian-Soureshjani A., Rafiee-Dastjerdi H., Naseri B., Hassanpour M., Khajehali J. (2023). Indoxacarb resistance in Iranian populations of *Tuta absoluta* (Lepidoptera: Gelechiidae): Cross-resistance, biochemical and molecular mechanisms. Pestic. Biochem. Physiol..

[175] van Lenteren J.C., Lanzoni A., Hemerik L., Bueno V.H.P., Bajonero Cuervo J.G., Biondi A., Burgio G., Calvo F.J., de Jong P.W., López S.N. (2021). The pest kill rate of thirteen natural enemies as aggregate evaluation criterion of their biological control potential of *Tuta absoluta*. Sci. Rep..

[176] Urbaneja A., González-Cabrera J., Arnó J., Gabarra R. (2012). Prospects for the biological control of *Tuta absoluta* in tomatoes of the Mediterranean basin. Pest Manag. Sci..

[177] Trdan S., Laznik Ž., Bohinc T. (2020). Thirty Years of Research and Professional Work in the Field of Biological Control (Predators, Parasitoids, Entomopathogenic and Parasitic Nematodes) in Slovenia: A Review. Appl. Sci..

[178] Rubio F.A., Montes F.C., Alpízar-Brenes G., Parra J.R.P., Jamielniak J.A., Lombardi Junior L.P., Vilches T.N. (2022). A predator-parasitoid mathematical model to describe the biological control of the tomato leafminer *Tuta absoluta*. Ecol. Complex..

[179] Akat O., Bayhan S. (2024). Determination of Predators and Parasitoids of *Tuta absoluta* (Lepidoptera: Gelechiidae) in Different Tomato Varieties Cultivated in Open Fields in Diyarbakır Province. Res. Agric. Sci..

[180] Ferracini C., Bueno V.H.P., Dindo M.L., Ingegno B.L., Luna M.G., Salas Gervassio N.G., Sánchez N.E., Siscaro G., van Lenteren J.C., Zappalà L. (2019). Natural enemies of *Tuta absoluta* in the Mediterranean basin, Europe and South America. Biocontrol Sci. Technol..

[181] Arnó J., Castañé C., Alomar O., Riudavets J., Agustí N., Gabarra R., Albajes R. (2018). Forty years of biological control in Mediterranean tomato greenhouses: The story of success. Isr. J. Entomol..

[182] Biondi A., Guedes R.N.C., Wan F.H., Desneux N. (2018). Ecology, Worldwide Spread, and Management of the Invasive South American Tomato Pinworm, *Tuta absoluta*: Past, Present, and Future. Annu. Rev. Entomol..

[183] Mansour R., Biondi A. (2021). Releasing natural enemies and applying microbial and botanical pesticides for managing *Tuta absoluta* in the MENA region. Phytoparasitica.

[184] Pérez-Hedo M., Gallego C., Roda A., Kostyk B., Triana M., Alférez F., Stansly P.A., Qureshi J., Urbaneja A. (2021). Biological traits of the predatory mirid Macrolophus praeclarus, a candidate biocontrol agent for the Neotropical region. Bull. Entomol. Res..

[185] Pérez-Hedo M., Riahi C., Urbaneja A. (2021). Use of zoophytophagous mirid bugs in horticultural crops: Current challenges and future perspectives. Pest Manag. Sci..

[186] Ismoilov K., Wang M., Jalilov A., Zhang X., Lu Z., Saidov A., Sun X., Han P. (2020). First Report Using a Native Lacewing Species to Control *Tuta absoluta*: From Laboratory Trials to Field Assessment. Insects.

[187] Basit A., Ullah F., Akhtar M.R., Humza M., Ghafar M.A., Hyder M., Haq I.U., Hou Y. (2025). Transforming *Tuta absoluta* Management: A Synergistic Approach Integrating Sustainability, Biological Control, and Biotechnological Innovations. Insects.

[188] Salas Gervassio N.G., Aquino D., Vallina C., Biondi A., Luna M.G. (2019). A re-examination of *Tuta absoluta* parasitoids in South America for optimized biological control. J. Pest Sci..

[189] Salas Gervassio N.G., Luna M.G., Minardi G.M., Sánchez N.E. (2019). Assessing inoculative releases of Pseudapanteles dignus (Hymenoptera: Braconidae) for the biological control of *Tuta absoluta* (Lepidoptera: Gelechiidae). Crop Prot..

[190] Arnó J., Molina P., Aparicio Y., Denis C., Gabarra R., Riudavets J. (2021). Natural enemies associated with *Tuta absoluta* and functional biodiversity in vegetable crops. BioControl.

[191] Hogg B.N., Bugg R.L., Daane K.M. (2011). Attractiveness of common insectary and harvestable floral resources to beneficial insects. Biol. Control.

[192] Laubertie E.A., Wratten S.D., Hemptinne J.-L. (2012). The contribution of potential beneficial insectary plant species to adult hoverfly (Diptera: Syrphidae) fitness. Biol. Control..

[193] Gonthier J., Arnó J., Romeis J., Collatz J. (2024). Insight into the host-specificity of a native and a newly introduced parasitoid of *Tuta absoluta* and prospect for biological control. Biol. Control..

[194] Labaude S., Griffin C.T. (2018). Transmission Success of Entomopathogenic Nematodes Used in Pest Control. Insects.

[195] Koppenhöfer A.M., Shapiro-Ilan D.I., Hiltpold I. (2020). Entomopathogenic Nematodes in Sustainable Food Production. Front. Sustain. Food Syst..

[196] Sajnaga E., Kazimierczak W. (2020). Evolution and taxonomy of nematode-associated entomopathogenic bacteria of the genera Xenorhabdus and Photorhabdus: An overview. Symbiosis.

[197] Lu D., Macchietto M., Chang D., Barros M.M., Baldwin J., Mortazavi A., Dillman A.R. (2017). Activated entomopathogenic nematode infective juveniles release lethal venom proteins. PLoS Pathog..

[198] Kamou N., Papafoti A., Chatzaki V., Kapranas A. (2024). Exploring the effects of entomopathogenic nematode symbiotic bacteria and their cell free filtrates on the tomato leafminer *Tuta absoluta* and its predator Nesidiocoris tenuis. J. Invertebr. Pathol..

[199] Ben Husin T.O., Port G.R. (2021). Efficacy of entomopathogenic nematodes against *Tuta absoluta*. Biol. Control.

[200] Kajuga J.N., Waweru B.W., Bazagwira D., Ishimwe P.M., Ndacyayisaba S., Mukundiyabo G.C., Mutumwinka M., Uwimana J.D., Toepfer S. (2025). Efficacy of Foliar Applications of Entomopathogenic Nematodes in the Management of the Invasive Tomato Leaf Miner *Phthorimaea absoluta* Compared to Local Practices Under Open-Field Conditions. Agronomy.

[201] Van Damme V.M., Beck B.K., Berckmoes E., Moerkens R., Wittemans L., De Vis R., Nuyttens D., Casteels H.F., Maes M., Tirry L. (2016). Efficacy of entomopathogenic nematodes against larvae of *Tuta absoluta* in the laboratory. Pest Manag. Sci..

[202] Samie F., Abbasipour H., Saeedizadeh A.J.R.d.l.S.E.A. (2023). Efficacy of three local isolates of entomopathogenic nematodes against the tomato leafminer, *Tuta absoluta* (Meyrick). Rev. De La Soc. Entomológica Argent..

[203] Li X., Liu X., Lu W., Yin X., An S. (2022). Application progress of plant-mediated RNAi in pest control. Front. Bioeng. Biotechnol..

[204] Hashmi M.H., Tariq H., Saeed F., Demirel U., Gökçe A., Merzendorfer H., Aksoy E., Bakhsh A. (2024). Harnessing plant-mediated RNAi for effective management of *Phthorimaea absoluta* by targeting AChE1 and SEC23 genes. Plant Stress.

[205] Hoang B.T.L., Fletcher S.J., Brosnan C.A., Ghodke A.B., Manzie N., Mitter N. (2022). RNAi as a Foliar Spray: Efficiency and Challenges to Field Applications. Int. J. Mol. Sci..

[206] Zhu K.Y., Palli S.R. (2020). Mechanisms, Applications, and Challenges of Insect RNA Interference. Annu. Rev. Entomol..

[207] Cooper A.M.W., Silver K., Zhang J., Park Y., Zhu K.Y. (2019). Molecular mechanisms influencing efficiency of RNA interference in insects. Pest Manag. Sci..

[208] Chen J., Peng Y., Zhang H., Wang K., Tang Y., Gao J., Zhao C., Zhu G., Palli S.R., Han Z. (2021). Transcript level is a key factor affecting RNAi efficiency. Pestic. Biochem. Physiol..

[209] Mendoza-Alatorre M., Julian-Chávez B., Solano-Ornelas S., Siqueiros-Cendón T.S., Torres-Castillo J.A., Sinagawa-García S.R., Abraham-Juárez M.J., González-Barriga C.D., Rascón-Cruz Q., Siañez-Estrada L.I. (2025). RNAi in Pest Control: Critical Factors Affecting dsRNA Efficacy. Insects.

[210] Rigano M.M., Scotti N., Cardi T. (2012). Unsolved problems in plastid transformation. Bioengineered.

[211] Kumar S. (2019). Biosafety and Ethical Issues in Genetic Engineering Research. Training Manual on Genetic Engineering: Principles and Practices.

[212] Roberts A.F., Devos Y., Lemgo G.N., Zhou X. (2015). Biosafety research for non-target organism risk assessment of RNAi-based GE plants. Front. Plant Sci..

[213] Zhao J.H., Liu Q.Y., Xie Z.M., Guo H.S. (2024). Exploring the challenges of RNAi-based strategies for crop protection. Adv. Biotechnol..

[214] Hernández-Soto A., Chacón-Cerdas R. (2021). RNAi Crop Protection Advances. Int. J. Mol. Sci..

[215] Zannou A.J., Arnó J., Romeis J., Collatz J. (2025). Compatibility of biocontrol agents with host plant resistance for management of the South American tomato pinworm *Phthorimaea absoluta*. Biol. Control.

[216] Tanga C.M., Onyango L.O., Malusi P., Subramanian S., Tenkouano A., Beesigamukama D. (2026). Insect oil-based biorationals as novel alternative against the invasive tomato leafminer [*Phthorimaea absoluta*] and fall armyworm [*Spodoptera frugiperda*]. J. Agric. Food Res..

[217] Zhang G., Wang H., Zhang Y., Xian X., Huang C., Liu W., Wan F. (2026). Performance and functional responses of the thelytokous and arrhenotokous strains of Neochrysocharis formosa to *Tuta absoluta*, a globally severe tomato pest. J. Integr. Agric..

[218] Aigbedion-Atalor P.O., Mohamed S.A., Hill M.P., Zalucki M.P., Azrag A.G.A., Srinivasan R., Ekesi S. (2020). Host stage preference and performance of *Dolichogenidea gelechiidivoris* (Hymenoptera: Braconidae), a candidate for classical biological control of *Tuta absoluta* in Africa. Biol. Control.

[219] Balzan M.V., Wäckers F.L. (2013). Flowers to selectively enhance the fitness of a host-feeding parasitoid: Adult feeding by *Tuta absoluta* and its parasitoid *Necremnus artynes*. Biol. Control.

[220] Abou El-Ela A.S., Dessoky E.S., Masry S., Arshad A., Munawar A., Qamer S., Abdelkhalek A., Behiry S.I., Kordy A. (2021). Plasticity in life features, parasitism and super-parasitism behavior of Bracon hebetor, an important natural enemy of Galleria mellonella and other lepidopteran host species. Saudi J. Biol. Sci..

[221] Soares M.A., Campos M.R., Passos L.C., Carvalho G.A., Haro M.M., Lavoir A.-V., Biondi A., Zappalà L., Desneux N. (2019). Botanical insecticide and natural enemies: A potential combination for pest management against *Tuta absoluta*. J. Pest Sci..

[222] Ingegno B.L., Ferracini C., Gallinotti D., Alma A., Tavella L. (2013). Evaluation of the effectiveness of *Dicyphus errans* (Wolff) as predator of *Tuta absoluta* (Meyrick). Biol. Control.

[223] Agboka K.M., Tonnang H.E.Z., Abdel-Rahman E.M., Odindi J., Mutanga O., Mohamed S.A. (2022). A Fuzzy-Based Model to Predict the Spatio-Temporal Performance of the *Dolichogenidea gelechiidivoris* Natural Enemy against *Tuta absoluta* under Climate Change. Biology.

[224] Chailleux A., Bearez P., Pizzol J., Amiens-Desneux E., Ramirez-Romero R., Desneux N. (2013). Potential for combined use of parasitoids and generalist predators for biological control of the key invasive tomato pest *Tuta absoluta*. J. Pest Sci..

[225] Chailleux A., Droui A., Bearez P., Desneux N. (2017). Survival of a specialist natural enemy experiencing resource competition with an omnivorous predator when sharing the invasive prey *Tuta absoluta*. Ecol. Evol..

[226] Alam A., Abbas S., Abbas A., Abbas M., Hafeez F., Shakeel M., Xiao F., Zhao C.R. (2023). Emerging trends in insect sex pheromones and traps for sustainable management of key agricultural pests in Asia: Beyond insecticides—A comprehensive review. Int. J. Trop. Insect Sci..

[227] Yang S., Cai Y., Miao Z. (2025). An improved and convenient new synthesis of the pheromone components of the tomato leafminer *Tuta absoluta*. RSC Adv..

[228] Jabamo T., Ayalew G., Goftishu M., Wakgari M. (2023). Integrated effect of insecticide and sex pheromone on the tomato leafminer, *Tuta absoluta* (Lepidoptera: Gelechiidae). Crop Prot..

[229] Wang M., Ismoilov K., Li H., Zhang X., Lu Z., Feng L.-k., Dai H.-J., Ye Z.-p., Biondi A., Desneux N. (2021). Polygyny of *Tuta absoluta* may affect sex pheromone-based control techniques. Entomol. Gen..

[230] Zhang G.-F., Zhang Y.-B., Zhao L., Wang Y.-S., Huang C., Lü Z.-C., Li P., Liu W.-C., Xian X.-Q., Zhao J.-N. (2023). Determination of Hourly Distribution of *Tuta absoluta* (Meyrick) (Lepidoptera: Gelechiidae) Using Sex Pheromone and Ultraviolet Light Traps in Protected Tomato Crops. Horticulturae.

[231] Shahini S., Bërxolli A., Kokojka F. (2021). Effectiveness of bio-insecticides and mass trapping based on population fluctuations for controlling *Tuta absoluta* under greenhouse conditions in Albania. Heliyon.

[232] Roda A., Brambila J., Barria J., Euceda X., Korytkowski C. (2015). Efficiency of Trapping Systems for Detecting *Tuta absoluta* (Lepidoptera: Gelechiidae). J. Econ. Entomol..

[233] Mangrio G.Q., Gilal A.A., Rajput L.B., Hajano J.-U.D., Gabol A.H. (2023). Performance of pheromone and light traps in monitoring and management of tomato leafminer, *Tuta absoluta* (Lepidoptera: Gelechiidae). J. Saudi Soc. Agric. Sci..

[234] Kadel J., Sah L., Devkota M., Colavito L., Norton G., Rajotte E., Muniappan R. (2018). Effectiveness of Different Types of Traps For Management of *Tuta absoluta* in Nepal. J. Plant Prot. Soc..

[235] Mahmoud Y., Ebadah I., Abd-Elrazik A., Abd-Elwahab T., Masry S. (2014). Efficiency of Different Colored Traps Baited with Pheromone in Capturing Tomato Adult Moth, *Tuta absoluta* (Meyrick) (Lepidoptera: Gelechiidae) during Summer Plantation. World Appl. Sci. J..

[236] Braham M. (2014). Sex Pheromone Traps for Monitoring the Tomato Leafminer, *Tuta absoluta*: Effect of Colored Traps and Field Weathering of Lure on Male Captures. Res. J. Agric. Environ. Manag..

[237] Désiré Anicet K., Tienebo E.-O., Armand A., Sory A., Yao A., Gadji A., Brou Y. (2025). Comparative Efficacy of Two Sex Pheromone Trap Types (Deltasan and Tutasan) for Monitoring and Mass Trapping of *Tuta absoluta* (Lepidoptera: Gelechiidae) in Tomato Crops in Cote d’Ivoire. Int. J. Plant Soil Sci..

[238] Solaiman R. (2016). Biological and Sex Pheromone Studies on Tomato Leaf Miner, *Tuta absoluta*, Meyrick (Lepidoptera: Gelechiidae) at Fayoum goveRnorate, Egypt. J. Plant Prot. Pathol..

[239] Desneux N., Han P., Mansour R., Arnó J., Brévault T., Campos M.R., Chailleux A., Guedes R.N.C., Karimi J., Konan K.A.J. (2022). Integrated pest management of *Tuta absoluta*: Practical implementations across different world regions. J. Pest Sci..

[240] Anugwom U.D., Audi A.H. (2026). Potential of pheromone attractants for managing insect pests of arable crops in Sub-Saharan Africa. J. Asia-Pac. Entomol..

[241] He Y., Ma L., Pu Q., Mao Z., Wang S., Wang T., Pu J., Ning J., Abou El-Ela A.S., Zhou W. (2024). Greenhouse trapping assessment and population dynamics of leaf miner *Tuta absoluta* (Meyrick) in E-Shan, Southwest China. Int. J. Pest Manag..

[242] Rwomushana I., Beale T., Chipabika G., Day R., Gonzalez-Moreno P., Lamontagne-Godwin J., Makale F., Pratt C., Tambo J. (2019). Tomato leafminer (Tuta absoluta): Impacts and Coping Strategies for Africa.

[243] Mawcha K.T., Kinyanjui G., Berhe D.H., Hategekimana A., Joelle K., Ndolo D. (2025). An overview of sustainable management strategies for *Tuta absoluta*. Int. J. Pest Manag..

[244] Han P., Desneux N., Becker C., Larbat R., Le Bot J., Adamowicz S., Zhang J., Lavoir A.-V. (2019). Bottom-up effects of irrigation, fertilization and plant resistance on *Tuta absoluta*: Implications for Integrated Pest Management. J. Pest Sci..

[245] Queiroz R.B., Lopes M.C., Costa T.L., da Silva R.S., Galdino T.V.S., Gontijo P.d.C., Martinez H.E.P., Picanço M.C. (2022). Influence of tomato plants nutritional status on the fitness and damage of *Tuta absoluta* (Lepidoptera: Gelechiidae). Agric. For. Entomol..

[246] Dong Y.-C., Han P., Niu C.-Y., Zappalà L., Amiens-Desneux E., Bearez P., Lavoir A.-V., Biondi A., Desneux N. (2018). Nitrogen and water inputs to tomato plant do not trigger bottom-up effects on a leafminer parasitoid through host and non-host exposures. Pest Manag. Sci..

[247] Mir M.S., Saxena A., Kanth R.H., Raja W., Dar K.A., Mahdi S.S., Bhat T.A., Naikoo N.B., Nazir A., Amin Z. (2022). Role of intercropping in sustainable insect-pest management: A review. Int. J. Environ. Clim. Change.

[248] Zarei E., Fathi S.A.A., Hassanpour M., Golizadeh A. (2019). Assessment of intercropping tomato and sainfoin for the control of *Tuta absoluta* (Meyrick). Crop Prot..

